# Molecular dissection of TatC defines critical regions essential for protein transport and a TatB–TatC contact site

**DOI:** 10.1111/j.1365-2958.2012.08151.x

**Published:** 2012-07-13

**Authors:** Holger Kneuper, Barbara Maldonado, Franziska Jäger, Martin Krehenbrink, Grant Buchanan, Rebecca Keller, Matthias Müller, Ben C Berks, Tracy Palmer

**Affiliations:** 1 Division of Molecular Microbiology, College of Life Sciences, University of DundeeDundee DD1 5EH, UK; 2 Department of Biochemistry, University of OxfordSouth Parks Road, Oxford OX1 3QU, UK; 3 Institute of Biochemistry and Molecular Biology, ZBMZStefan-Meier-Strasse 17, D-79104 Freiburg, Germany

## Abstract

The twin arginine transport (Tat) system transports folded proteins across the prokaryotic cytoplasmic membrane and the plant thylakoid membrane. TatC is the largest and most conserved component of the Tat machinery. It forms a multisubunit complex with TatB and binds the signal peptides of Tat substrates. Here we have taken a random mutagenesis approach to identify substitutions in *Escherichia coli* TatC that inactivate protein transport. We identify 32 individual amino acid substitutions that abolish or severely compromise TatC activity. The majority of the inactivating substitutions fall within the first two periplasmic loops of TatC. These regions are predicted to have conserved secondary structure and results of extensive amino acid insertion and deletion mutagenesis are consistent with these conserved elements being essential for TatC function. Three inactivating substitutions were identified in the fifth transmembrane helix of TatC. The inactive M205R variant could be suppressed by mutations affecting amino acids in the transmembrane helix of TatB. A physical interaction between TatC helix 5 and the TatB transmembrane helix was confirmed by the formation of a site-specific disulphide bond between TatC M205C and TatB L9C variants. This is the first molecular contact site mapped to single amino acid level between these two proteins.

## Introduction

In bacteria and archaea two general pathways mediate the export of proteins across the cytoplasmic membrane. The Sec pathway, which is the predominant route of protein export in most prokaryotes, uses a threading mechanism to transport unfolded proteins (Van den Berg *et al*., 2004; Driessen and Nouwen, 2008). The twin arginine transport (Tat) pathway, by contrast, exports folded proteins (reviewed in Cline and Theg, 2007; Frobel *et al*., 2012; Palmer and Berks, 2012). Proteins are targeted to the respective pathway by means of specific N-terminal signal peptides that are usually cleaved from the substrate protein during transport. Signal peptides that target to the Tat machinery have several features that distinguish them from Sec signals, most notably the presence of a conserved S-R-R-x-F-L-K ‘twin arginine’ motif where the arginines are almost invariant (Berks, 1996).

In the model bacterium *Escherichia coli* the Tat machinery comprises three main components; TatA, TatB and TatC (Bogsch *et al*., 1998; Sargent *et al*., 1998; 1999; Weiner *et al*., 1998). A fourth protein, TatE, is a minor component of the Tat machinery with an identical function to TatA (Sargent *et al*., 1998; Jack *et al*., 2001). Orthologues of TatA, TatB and TatC are found in most Gram-negative bacteria and in the thylakoid membranes of plant chloroplasts (Settles *et al*., 1997). However, some Gram-positive bacteria and archaea lack a TatB component and require only TatA and TatC proteins for functional Tat transport (Jongbloed *et al*., 2004; 2006).

TatB and TatC form a hetero-oligomeric membrane-bound complex (Bolhuis *et al*., 2001). The exact number of subunits in the complex is not known but current estimates suggest between six and eight copies of each protein are present (Bolhuis *et al*., 2001; de Leeuw *et al*., 2002; Oates *et al*., 2003; Richter and Bruser, 2005; Tarry *et al*., 2009). One of the major roles of the TatBC complex is the recognition of substrate proteins. It has been shown to interact with both precursor proteins and isolated signal peptides (Cline and Mori, 2001; de Leeuw *et al*., 2002; Alami *et al*., 2003; Richter and Bruser, 2005; Tarry *et al*., 2009). Photo-affinity cross-linking studies and genetic experiments have indicated that TatC is the primary recognition site of the twin arginine motif, while TatB interacts further along the signal peptide and with the mature domain of substrates (Alami *et al*., 2003; Gerard and Cline, 2006; Kreutzenbeck *et al*., 2007; Strauch and Georgiou, 2007; Zoufaly *et al*., 2012). The presence of multiple copies of each subunit is reflected in the observation that the TatBC complex is multivalent and can bind more than one substrate molecule at a time (Tarry *et al*., 2009; Ma and Cline, 2010). However, multivalency of TatBC is unlikely to be a strict requirement for operation of the Tat pathway because TatBC complexes where at least half of the TatC proteins were inactivated by point mutation retained their activity (Maldonado *et al*., 2011a).

Most current models for Tat transport propose that during transport small, possibly tetrameric units of TatA associate with a precursor-loaded TatBC complex, leading to the polymerization of TatA into much larger oligomers (Dabney-Smith *et al*., 2006; Leake *et al*., 2008; Dabney-Smith and Cline, 2009). This is supported by *in vitro* cross-linking experiments using isolated thylakoids and *E. coli* membrane vesicles and by *in vivo* evidence examining the behaviour of a fluorescent TatA fusion protein (Alami *et al*., 2003; Dabney-Smith *et al*., 2006; Leake *et al*., 2008; Dabney-Smith and Cline, 2009). The large TatBC-induced oligomers of TatA facilitate transport of substrates, and TatA complexes isolated in detergent solution form large ring-shaped structures, consistent with TatA forming the protein conducting channel (Gohlke *et al*., 2005).

Current knowledge about the Tat transport pathway is limited by a lack of high resolution structural information on the TatBC components. Disulphide cross-linking results have led to the proposal that the monotopic membrane protein TatB forms a homo-oligomeric ring in the centre of the TatBC complex, with TatC, which has six transmembrane domains, arranged as a homo-oligomer at the periphery (Lee *et al*., 2006; Punginelli *et al*., 2007). A site-specific photo-cross-linker introduced into the cytoplasmic N-terminus or second periplasmic loop of TatC cross-links to TatB, revealing the presence of contact sites on both sides of the membrane (Zoufaly *et al*., 2012). Site-directed mutagenesis studies of highly conserved residues in TatC proteins have identified a few residues that are absolutely required to support TatC function (Allen *et al*., 2002; Buchanan *et al*., 2002; Barrett and Robinson, 2005; Holzapfel *et al*., 2007; Punginelli *et al*., 2007). Most of these residues cluster at the cytoplasmic side of the membrane, with several of them in a loop region between transmembrane domains two and three. Site-specific photo-cross-linkers introduced into this loop region or in the cytoplasmic N-terminus of TatC cross-link with Tat precursors (Zoufaly *et al*., 2012), and the same cytoplasmic loop is also a hot-spot for the isolation of mutations that suppress the transport defect of signal peptides that carry a normally inactivating twin lysine substitution of the twin arginines (Strauch and Georgiou, 2007). Other suppressors of inactivating signal sequence mutations map to less conserved cytoplasmic regions of TatC (Kreutzenbeck *et al*., 2007).

In this study we have taken an unbiased approach to identify residues required for the activity of *E. coli* TatC by screening a random mutant library for *tatC* alleles that inactivate Tat transport. Our results unexpectedly show that the periplasmic regions of TatC, which show little sequence conservation, are particularly intolerant to substitution, probably due to conserved secondary structure in these parts of the protein. In addition we show that substitutions at some positions within transmembrane helix 5 also lead to inactivation, and that at least one of these can be suppressed by compensatory mutations in the transmembrane helix of TatB. A direct interaction between these regions of TatC and TatB is demonstrated by the detection of a site-specific disulphide cross-link between these regions of the two proteins.

## Results

### Isolation of random substitutions that inactivate the function of *E. coli* TatC

Previous studies which investigated the role of individual amino acids in *E. coli* TatC by targeting highly conserved residues identified very few residues critical for TatC activity (Allen *et al*., 2002; Buchanan *et al*., 2002; Barrett and Robinson, 2005). Moreover, conflicting phenotypes were reported for the same mutations in different studies. These discrepancies may be due to differences between experiments in the expression levels and relative stoichiometries of the Tat components, the substrates used, and the sensitivity of assays used to assess Tat activity. To get a more complete picture of regions within *E. coli* TatC that are critical for transport we randomly mutagenized the *tatC* gene and screened for mutations that resulted in loss of Tat function.

To construct a random library of *tatC* mutations, the wild-type *tatC* allele from plasmid pTAT1d that harbours the *tatABC* operon under control of the *tat* promoter (Maldonado *et al*., 2011b; Fig. S1) was amplified using error-prone PCR. The mutagenized products were subsequently ligated back into pTAT1d to replace the wild-type *tatC* allele. Transformation into ultracompetent *E. coli* cells resulted in a mutant library of approximately 600 000 individual clones with an average error rate of 0.3% (approximately 2.5 ± 1.5 errors per *tatC* gene) as determined by sequencing of 20 random clones.

This library was subsequently screened for loss-of-function mutations in *tatC* using a previously described positive selection protocol based on the export of chloramphenicol acetyltransferase (CAT) fused to the *E. coli* TorA Tat signal peptide (Maldonado *et al*., 2011b). In the presence of a fully functional Tat system, the fusion protein is exported from the cytoplasm, resulting in cells that are sensitive to chloramphenicol. On the other hand, strains harbouring mutations that impede or abolish Tat transport, and thus retain CAT in the cytoplasm, are resistant to chloramphenicol. Since this test alone does not distinguish between complete and partial inhibition of the Tat system, mutants identified by growth on chloramphenicol were further screened for absence of growth in the presence of 2% SDS. *E. coli* strains completely inactivated for the Tat pathway fail to grow in the presence of 2% SDS due to their inability to export two Tat-dependent amidase enzymes that are involved in cell wall remodelling (Buchanan *et al*., 2002; Ize *et al*., 2003). Thus under these screening conditions, only mutants with a fully inactive Tat system are unable to grow.

The plasmid-encoded mutant library was transformed into an *E. coli* strain which lacks the chromosomal *tat* genes and produces TorAss-CAT. A total of approximately 60 000 independent clones were screened. Of these, around 10% were able to grow on solid media containing 100 µg ml^−1^ chloramphenicol, which is broadly similar to what we observed when screening an analogous library for inactivating *tatB* mutations (Maldonado *et al*., 2011b). From these chloramphenicol resistant clones, 404 were randomly selected and replica-spotted onto LB plates containing either 100 µg ml^−1^ chloramphenicol or 2% SDS. Of these, 306 showed growth on chloramphenicol and were also unable to grow on 2% SDS, consistent with complete inactivation of Tat transport. The *tatC* genes from these clones were amplified by colony PCR and sequenced to identify the inactivating mutations. Nearly two-thirds of the sequenced clones (203 out of 306) contained either a frame shift or a premature stop codon within *tatC* (not shown). Such mutations were present at a low abundance in the original library (two stop codon mutations and no frame shifts from 20 clones sequenced) but were clearly enriched during the screening process. Sequencing of the remaining 103 mutants revealed the presence of between one and eight mutations distributed throughout the *tatC* sequence. A complete list of the substitutions found in these 103 clones is given in Table S1.

### Identification of single inactivating mutations in *tatC*

As fewer than 10% of the 103 clones isolated from the screen contained single amino acid changes, we additionally selected some of the multiple mutations from our screen and constructed the individual amino acid substitutions. In doing so we selected only those amino acid changes that lay between amino acid positions E_15_ through to L_225_ since multiple sequence alignment indicates that this is the most conserved part of TatC proteins (Buchanan *et al*., 2002). Mutations were introduced into plasmid pTAT101, a very low copy number plasmid that carries the wild-type *tatABC* operon under control of the *tat* promoter. This should eliminate any effects caused by strong overproduction of the Tat components. The TatC level produced from plasmid pTAT101 was approximately four times that of chromosomally produced TatC, as judged from immunoblotting of isolated membrane fractions (Fig. S2).

**Fig. 1:**
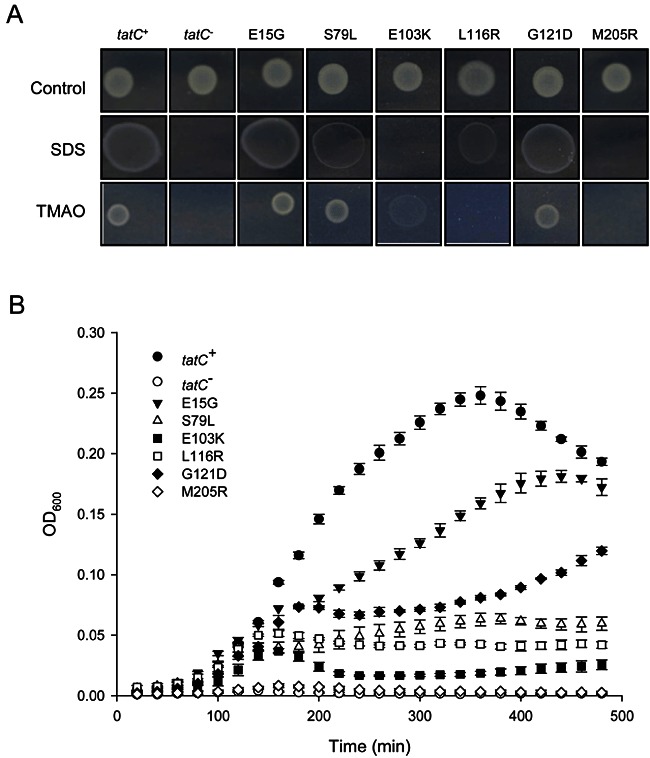
Phenotypic characterization of representative examples of TatC single amino acid variants. A. Spot tests of strain DADE (Δ*tatABCD*, Δ*tatE*) harbouring an empty plasmid vector pTH19kr (*tatC*^-^) or the same vector encoding TatA, TatB and either wild-type TatC (*tatC*^+^) or variants carrying the indicated amino acid substitution. Strains were spotted onto LB medium (Control) or LB medium containing 2% SDS and incubated aerobically overnight. Strains were also spotted onto M9 medium containing glycerol and TMAO and incubated for 3 days under anaerobic conditions. B. Growth curves of the same strain and plasmid combinations in LB liquid medium containing 0.5% SDS. Growth curves were recorded for 10 h in 96-well plates (200 µl volume) without shaking. The average and standard deviation of three replicates are shown.

We dissected out each of the double mutations to give the corresponding single mutations and in addition dissected many of our triple mutations and some of our multiple mutations (Table S1). Furthermore, we also re-constructed each of the single mutations isolated from the screen (Table S1) in this background. In total, 76 single amino acid exchanges were introduced into pTAT101-encoded TatC (these are listed in Table 1). Each of these mutant plasmids was transformed into the Δ*tatABCD*Δ*tatE* strain, DADE, and the activity of the Tat machinery was initially assessed using two phenotypic growth tests that screen for the export of native Tat substrates. The first of these is the ability to grow in the presence of SDS, as described above. The second is anaerobic growth of *E. coli* with trimethylamine-*N*-oxide (TMAO) as sole electron acceptor, which relies upon the ability of the cell to export the Tat substrates TMAO reductase and dimethylsulphoxide reductase (Sargent *et al*., 1998; Weiner *et al*., 1998). Examples of the behaviour of some of the strains harbouring mutated *tatC* genes are shown in Fig. 1A.

**Table 1:** Single amino acid substitutions introduced into TatC in this study

E15G	L49S	V64E	F94S	L111P	V145E	V198D
L16P	L53P	S66P	P97T	L116P	S148P	G204R
R17H	L53S	P67S	A98V	L116R	D150G	M205K
L20P	P54L	F68S	L99P	G121D	D150V	M205R
C23R	M59K	T70R	L99Q	F130S	I151T	P209L
L34P	I60N	L74P	Y100C	P131L	Y154H	P210R
Y42N	A61T	S79L	E103G	A133V	F157Y	S214P
S46F	A61V	L82Q	E103K	L137H	A160V	Q215R
A47T	T62A	P85L	R104C	A141E	M163K	A219E
P48L	D63V	Q90R	R105C	E143G	S168P	L225P
P48S	V64A	F94L	P109A	G144R	D188V	

Of the 76 single *tatC* mutants tested, 24 failed to support detectable growth in either of these phenotypic tests (listed in Table 2). Four of the TatC variants (L20P, L99Q, L116R and I151T; Fig. 1A; Table 2) supported marginal growth in the presence of SDS but did not allow any growth on TMAO selective media, while a further four variants (S46F, E103G, E103K and M205K; Fig. 1A; Table 2) did not support growth in the presence of SDS but allowed some poor growth with TMAO. The remaining mutations all allowed detectable growth on both types of media, indicating that the TatC proteins all retained at least some level of functionality.

**Table 2:** Amino acid substitutions that inactivate the function of TatC

L16P	I60N	F94S	L137H	M205K^b^
L20P^a^	D63V	L99P	V145E	M205R
C23R	V64E	L99Q^a^	S148P	Q215R
S46F^b^	S66P	E103G^b^	D150G	L225P
P48L	F68S	E103K^b^	D150V	
P48S	T70R	L116R^a^	I151T^a^	
M59K	L74P	P131L	G204R	

a Mutations in TatC that support growth on SDS but not TMAO.

b Mutations in TatC that support growth on TMAO but not on SDS.

Note that a V147E was constructed fortuitously while we were constructing the V145E substitution. This also fully inactivated the function of TatC.

**Fig. 2:**
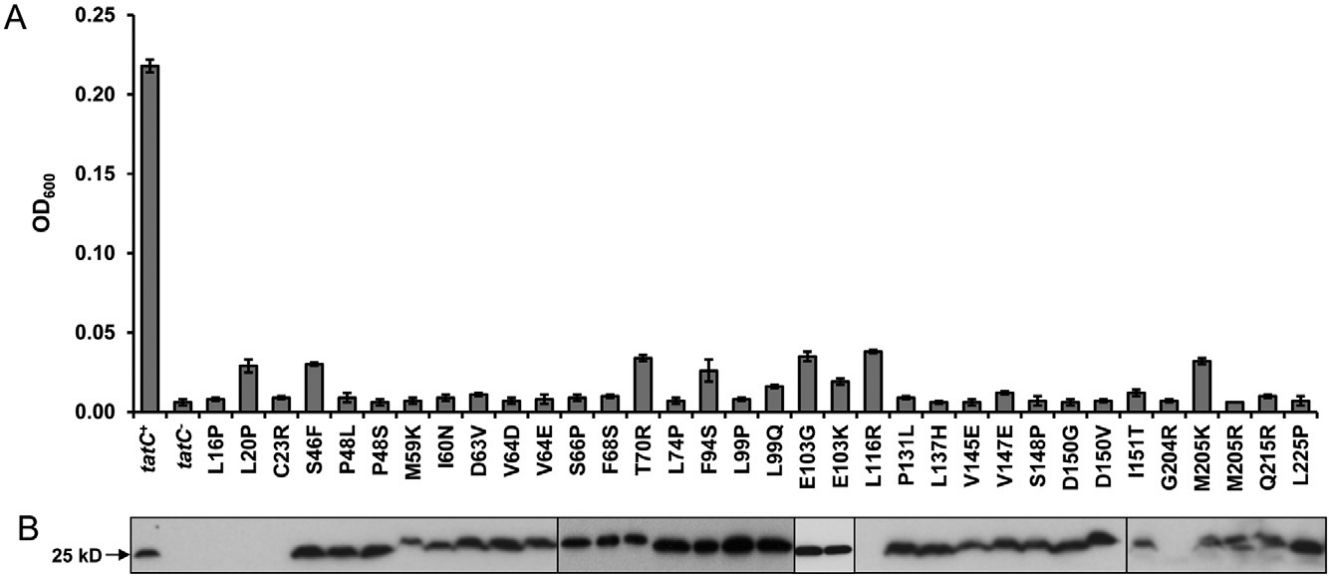
Analysis of inactive and poorly active single amino acid variants of TatC. A. Growth of strain DADE (Δ*tatABCD*, Δ*tatE*) harbouring an empty plasmid vector pTH19kr (*tatC*^-^) or the same vector encoding TatA, TatB and either wild-type TatC (*tatC*^+^) or variants carrying the indicated amino acid substitution in LB liquid medium containing 0.5% SDS. Growth was recorded in 96-well plates (200 µl volume) without shaking, and the optical density (600 nm) of each the culture after 6 h was plotted. The average and standard deviation of three replicates are shown. B. Analysis of variant TatC proteins by Western blotting. Urea-washed membrane fractions (40 µg) of the same strain and plasmid combinations were analysed by immunoblotting using anti-TatC antiserum.

Since the growth tests are largely qualitative, we developed a more quantitative assessment of growth in the presence of SDS using liquid growth assays. Growth curves in LB medium containing 0.5% SDS were recorded in a high-throughput 96-well format, and representative examples are shown in Fig. 1B. By following growth over several hours it became apparent that growth defects can be seen that were not picked up by assessing growth on solid media. For example, strains producing the E15G and G121D TatC variants grew more slowly than the same strain producing wild-type TatC, while the strain harbouring the S79L variant, which also showed growth in plate tests, grew only very poorly in the liquid growth assay. The M205R variant of TatC did not allow any growth, and was indistinguishable from a strain lacking *tatC*. Growth curves for *E. coli* strains producing each of the inactive TatC variants listed in Table 2 were subsequently recorded in SDS-containing medium. The optical density (600 nm) after 6 h of growth was chosen as an indicator of growth, as this was the time point by which the strain producing wild-type TatC reached the end of the exponential growth phase. As expected, the results of the growth assays, shown in Fig. 2A, indicated that the growth of strains producing the majority of these TatC variants was indistinguishable from that of the negative control (strain DADE carrying the empty plasmid vector pTH19kr).

We next examined whether any of the 32 single amino acid substitutions listed in Table 2 that abolished or severely affected the activity of TatC had any detectable effect on TatC levels in the cytoplasmic membrane. Membrane fractions of strain DADE producing wild-type TatA and TatB along with either wild-type TatC or each of the TatC variants were prepared, subjected to SDS-PAGE and analysed for the presence of TatC by immunoblotting. The membrane fraction of DADE carrying the empty plasmid vector, pTH19kr, served as a negative control. As shown in Fig. 2B, TatC could clearly be detected at approximately similar levels for all but five of the TatC variants. TatC proteins containing the L16P, L20P, C23R, L116R or G204R substitutions could not be detected, suggesting that these proteins were either completely absent, or that the antibody binding epitope (which is at the extreme C-terminus of TatC) had been proteolysed. Some of the TatC variants also showed slightly anomalous migration, in particular the M59K variant, which migrated more slowly than the wild-type protein.

**Fig. 3:**
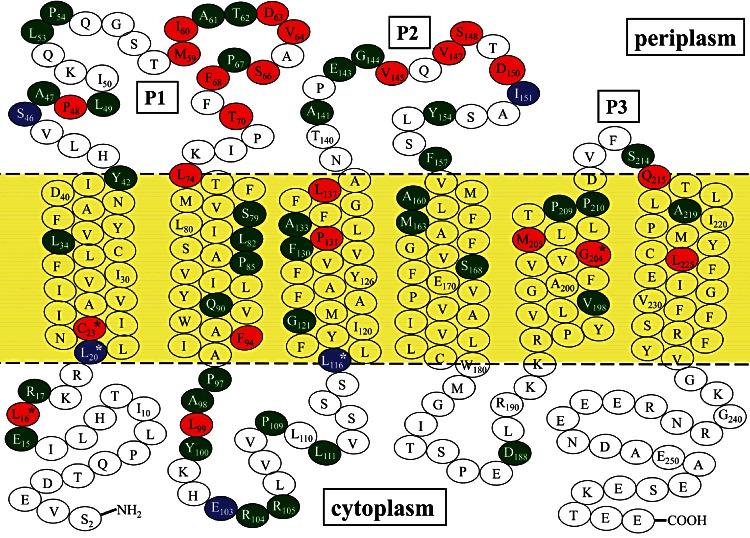
Location of single amino acid exchanges in *E. coli* TatC analysed in this study. The positions of the predicted transmembrane helices are indicated (predicted using TMHMM 2.0; http://www.cbs.dtu.dk/services/TMHMM/), the periplasmic loops (P1, P2 and P3 respectively) are labelled and residues targeted for single amino acid substitutions are highlighted: in *green*, no apparent effect on TatC activity in phenotypic growth tests; in *red*, inactivating mutation, based on the absence of Tat function in both phenotypic growth tests; in *blue*, mutation that severely affects the activity of TatC (i.e. that permitted growth in only one of the two phenotypic tests for Tat function). Residues marked with an asterisk indicate where amino acid substitution resulted in non-detectable TatC protein.

In summary, 24 of the 76 individual amino acid substitutions constructed in TatC completely inactivated TatC function, while a further eight severely compromised TatC activity by preventing growth in one of the two Tat-selective phenotypic growth tests. The location of these mutations within the predicted secondary structure of TatC is shown in Fig. 3.

### The periplasmic loops of TatC are hotspots for inactivating mutations

Mutations inactivating TatC function were isolated throughout the protein sequence, with more inactivating substitutions in the predicted membrane-extrinsic regions of the protein than in the transmembrane domains. Previously mutations that severely affect the function of TatC have been identified in or close to the first cytoplasmic loop, including alanine substitutions of positions F94, P97, L99, Y100 and E103 (Buchanan *et al*., 2002; Holzapfel *et al*., 2007). In our screen we also isolated five mutations critically affecting TatC function that fell within this region, including an F94S substitution, and two independent substitutions at L99 (to P or Q) and E103 (to G or K). This underscores the importance of this region of TatC, and confirms the validity of our random mutagenesis and screening approach.

Surprisingly, half of the inactivating substitutions we isolated were in periplasmic regions of TatC (Fig. 3). There is very little conservation of sequence or even length in the first two extrinsic loops of TatC, for example, the first periplasmic loop (P1) varies in length between 14 amino acids (*Staphylcoccus aureus* TatC; Fig. S3) and 248 amino acids (*Pirellula staleyi* ATCC27377), while the second periplasmic loop (P2) varies in length between 19 amino acids (e.g. *E. coli* TatC) and 183 amino acids (*Myxococcus xanthus* TatC). The P1 loop contains a moderately conserved proline residue (P48 in the *E. coli* sequence) that when mutated to alanine blocks Tat function and the formation or stability of the TatBC complex (Allen *et al*., 2002; Barrett *et al*., 2005; Barrett and Robinson, 2005). No inactivating mutations have previously been identified in the P2 loop. By contrast, the third periplasmic loop, P3, is always extremely short, with a highly conserved Pro-Asp pairing (P210–D211 for *E. coli* TatC), with substitution of the Asp residue with Ala abolishing the function of *E. coli* TatC (Buchanan *et al*., 2002).

**Fig. 4:**
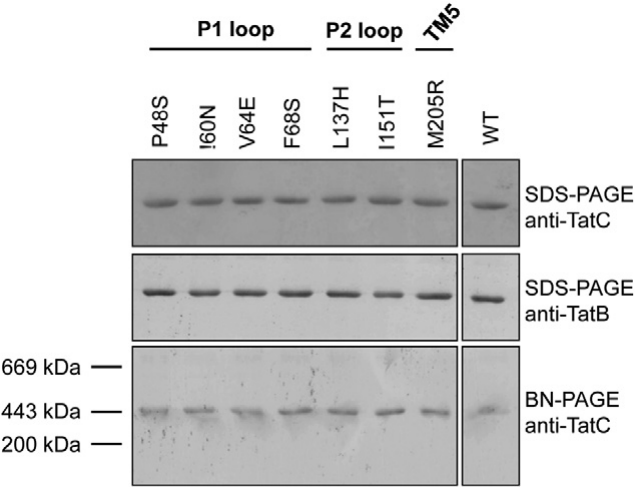
Transport inactive TatC variants can still form complexes with TatB. TatBC complexes were nickel-affinity purified from digitonin-solubilized membrane fractions of *E. coli* DADE producing wild-type TatA and TatB alongside hexahistidine-tagged TatC wild-type or variants P48S, I60N, V64E, F68S, L137H, I151T or M205R. Eluted proteins were separated by SDS-PAGE or BN-PAGE and analysed by immunoblotting using anti-TatC or anti-TatB antiserum, as indicated.

In our random screen we isolated substitutions that completely inactivated TatC that affected eight residues in P1 (Table 2; Fig. 3), and a further substitution (S46F) that severely compromised TatC activity by preventing growth on SDS (but not on TMAO). Two of the inactivating substitutions affected P48, replacing it with leucine or serine. Given that a P48A substitution in TatC resulted in aberrant formation or instability of the TatBC complex (Barrett *et al*., 2005) we tested complex formation for a selection of the inactivating P1 loop substitutions. The P48S, I60N, V64E and F68S-substituted TatC proteins were co-produced as his-tagged variants together with wild-type TatA and TatB. After solubilizing membranes with 1% digitonin, tagged TatC proteins were bound to magnetic Ni-charged beads, washed and eluted with imidazole. The presence of the TatC variants in the elution fraction was confirmed by SDS-PAGE and Western blotting. In each case TatB co-purified at similar levels to that co-purifying with the wild-type TatC variant (Fig. 4). The sizes of the purified TatC-containing complexes were analysed by blue native PAGE and immunoblotting with anti-TatC antiserum. In each case one strongly reacting band was detected that migrated close to the 443 kDa marker, at the same position as the wild-type TatBC complex (Fig. 4). We conclude that the assembly and stability of the TatBC complex is not affected by any of these substitutions.

**Fig. 5:**
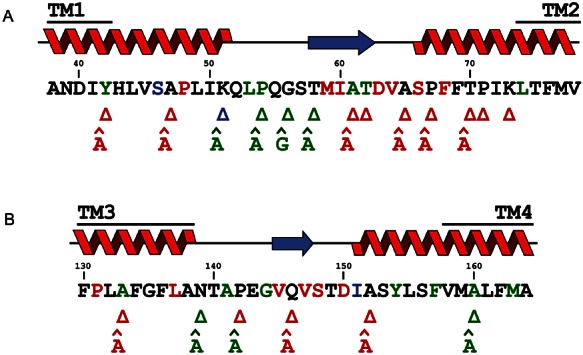
Mutagenesis of the P1 and P2 loops of TatC. The amino acid sequence of (A) the P1 and (B) P2 loop is shown. Residues in green, blue or red highlight the positions of single amino acid exchanges identified through the random mutagenesis screen, the colour coding is as for Fig. 3. Above the sequence is the predicted secondary structure, with α-helix shown in red and β-sheet in blue. The predicted boundaries of the transmembrane helices are also shown. The positions of single amino acid deletions (indicated by the Δ symbol) and single alanine insertions (indicated by A) are given below the sequence, with colour-coding to indicate the effect of the mutation where *green* signifies no apparent effect on TatC in phenotypic growth tests; *red* signifies inactivating mutation, based on the absence of Tat function in both phenotypic growth tests and *blue* signifies a mutation that severely affects the activity of TatC (i.e. that permitted growth in only one of the two phenotypic tests for Tat function). Note that the V147E substitution was constructed fortuitously while we were constructing the V145E substitution.

Despite the lack of sequence or length conservation of the P1 loop, structural prediction programmes, for example, JPred3 (Cole *et al*., 2008), suggest that this loop contains regions of conserved secondary structure. As shown in Fig. 5A, the start of the loop region (which is predicted to be at or around H43 for *E. coli* TatC) is strongly predicted to be a polar α-helical stretch, extending as far as Q53. There is a predicted region of β-sheet leading into a second polar α-helical stretch that becomes hydrophobic at or around L74, where it is predicted to form transmembrane segment 2. Larger TatCs which have extended P1 loops in general appear to have the additional amino acid stretches inserted in the region between these secondary structural elements (not shown). Mapping the positions of the mutations onto the predicted secondary structure revealed that most of the inactivating mutations coincided with the predicted secondary structure elements, suggesting that the conserved structure of the P1 loop may be critical for TatC function.

**Fig. 6:**
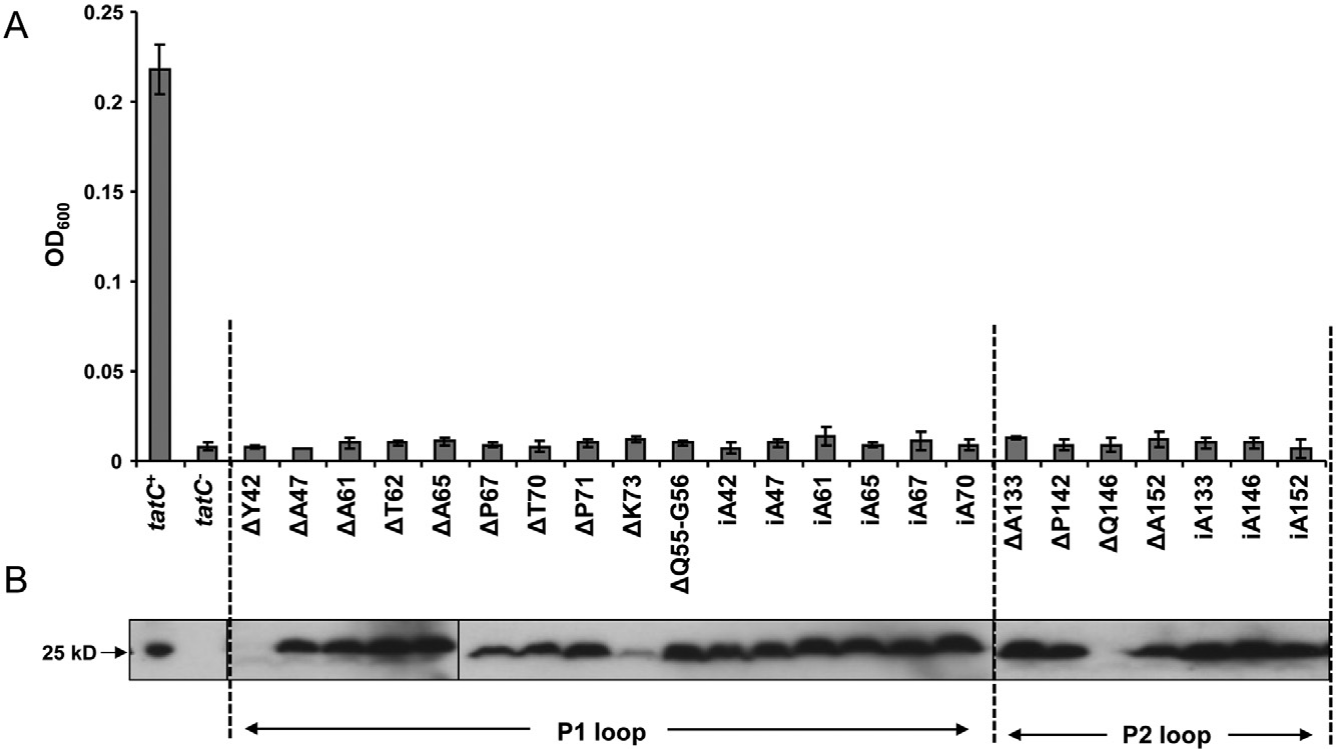
Amino acid insertion and deletion mutations in the P1 and P2 loops inactivate the function of TatC. A. Growth of strain DADE (Δ*tatABCD*, Δ*tatE*) harbouring an empty plasmid vector pTH19kr (*tatC*^-^) or the same vector encoding TatA, TatB and either wild-type TatC (*tatC*^+^) or variants carrying the indicated amino acid insertion or deletion in LB liquid medium containing 0.5% SDS. Growth was recorded in 96-well plates (200 µl volume) without shaking, and the optical density (600 nm) of each culture after 6 h was plotted. The average and standard deviation of three replicates are shown. B. Analysis of variant TatC proteins by Western blotting. Urea-washed membrane fractions (40 µg) of the same strain and plasmid combinations were analysed by immunoblotting using anti-TatC antiserum.

To further probe structure-function correlates in the P1 loop we constructed a series of single amino acid deletions and insertions spanning the entire length of this loop (listed in Table 3) in plasmid pTAT101. Thirteen single amino acid deletions and 10 insertions were constructed as shown in Fig. 5A, avoiding residues that we had already found to be inactivated by substitution in our random mutagenesis screen. In most cases insertions were of an alanine residue with the exception of position G56 where a glycine was inserted. The insertion and deletion mutants were characterized for TatC activity using the phenotypic tests described above. Those TatC variants that did not support any detectable growth with TMAO or SDS, shown in red font in Fig. 5A, were subsequently analysed for growth in liquid medium containing SDS, and the level of TatC in the membrane fraction was detected by Western blotting (Fig. 6). Membrane-bound TatC could easily be detected for each of the inactive variants with the exception of the Y42 deletion, where no protein was observed, and the K73 deletion, where the TatC level was very low (Fig. 6B). Taken together these results indicate that single amino acid insertions or deletions were tolerated only in a small central region of the P1 loop covering residues K51 to T58, coinciding with the short region predicted to be devoid of secondary structure (Fig. 5A). By contrast, the largest part of the periplasmic loop, containing the predicted α-helical and β-sheet stretches, proved sensitive to single amino acid insertions and deletions, suggesting that these structural elements are essential for TatC function.

**Table 3:** Insertion and deletion mutations constructed in the P1 and P2 regions of TatC

ΔY42	ΔG56	iA65	iA131	iA152
iA42	iG56	ΔP67	ΔN139	ΔA160
ΔA47	ΔT58	iA67	iA139	iA160
iA47	iA58	ΔT70	ΔP142	iGG56
ΔK51	ΔA61	iA70	iA142	ΔQ55-G56
iA51	iA61	ΔP71	ΔQ146	ΔG56-T57
ΔP54	ΔT62	ΔK73	iA146	
iA54	ΔA65	ΔA131	ΔA152	

Insertions are prefixed by ‘i’, for example, iA42 indicates insertion of an alanine at residue 42. Note that iGG56 has two glycines inserted at residue 56. Deletions are prefixed by Δ, so ΔY42 indicates deletion of the tyrosine residue at position 42. ΔQ55-G56 and ΔG56-T57 are deletions of two consecutive residues.

We further introduced double amino acid deletions covering residues Q55-G56 and G56-S57 respectively, as well as an insertion of two additional glycine residues after position Q55, to test whether more extended length alterations were tolerated in this permissive region of the P1 loop. While the G56-S57 deletion and the double glycine insertion did not abolish TatC function, deletion of residues Q55-G56 completely inactivated TatC even thoughthis substitution did not affect membrane insertion or apparent stability of the protein (Fig. 6B). We conclude that almost the entire length of the P1 loop is sensitive to mutagenesis, but particularly those regions corresponding to conserved secondary structural elements.

The random mutagenesis screen identified five substitutions of four residues in the P2 loop that also completely inactivated TatC. In addition we fortuitously isolated a further TatC inactivating substitution (V147E; Fig. 2) while we were constructing the V145E variant. To ascertain whether amino acid substitutions in or close to the P2 loop affected formation or stability of the TatBC complex, we selected the L137H and I151T TatC proteins and co-produced them as his-tagged variants together with wild-type TatA and TatB from an overexpression plasmid. In each case approximately wild-type levels of TatB was observed to co-purify with the his-tagged TatC proteins from digitonin-solubilized membranes, and Blue-Native polyacrylamide gel electrophoresis (BN-PAGE) analysis in each case showed a single complex which migrated at the same position as the wild-type TatBC complex (Fig. 4). We conclude that the assembly or stability of the TatBC complex is not grossly affected by either of these substitutions.

Analysis of potential secondary structural elements within the *E. coli* TatC P2 loop (Fig. 5B), suggests that the α-helical stretch of TM3 ends at or close to A138 and that there is a short region of predicted β-sheet leading into a polar α-helical stretch that becomes hydrophobic at or around F157 where it is predicted to start transmembrane segment 4. We therefore undertook an insertion and deletion analysis of the P2 loop, selecting six different locations from residue A133 to A160 (Table 3; Fig. 5B). Phenotypic screening indicated that single amino acid deletions or alanine insertions were tolerated at positions N139 and A160, as was an alanine insertion at position P142. By contrast, insertions and deletions at positions A133, Q146 and A152 and a deletion of P142 each resulted in an inactive TatC (Fig. 6A). TatC was detected in the membrane fraction for all of the inactive variants with the exception of the Q146 deletion (Fig. 6B). These data indicate that the P2 loop is sensitive to mutagenesis, particularly within and C-terminal to the predicted short stretch of β-sheet.

### An inactivating mutation in TatC transmembrane helix 5 can be suppressed by mutations in the transmembrane domain of TatB

We obtained surprisingly few inactivating mutations that fell within the transmembrane domains of TatC (Fig. 3). Some of these were substitutions of leucine to proline residues (e.g. L20P, L74P, L225P) and one was a proline to leucine substitution (P131L). Proline residues play very specific roles in transmembrane helices, introducing kinks and influencing helical packing (Cordes *et al*., 2002; Meruelo *et al*., 2011), and therefore mutations that substitute or introduce prolines might be expected to severely affect TatC function. Interestingly, however, we isolated consecutive inactivating substitutions at positions 204 and 205, located in transmembrane helix 5. This helix is unusual because it is predicted to be much shorter than the other five TMs of TatC (e.g. TMHMM predicts each of the TMs of *E. coli* TatC to comprise 22 amino acids with the exception of TM5, which is predicted to be 17 aa long). These substitutions were not to proline residues but instead introduced basic amino acids into the transmembrane domain (G204R, M205K, M205R). While G204R TatC was clearly unstable (Fig. 2B), the M205K and M205R variants were present in the membrane at close to wild-type levels (Fig. 2B) and the M205R mutation did not affect the assembly or stability of the TatBC complex (Fig. 4).

Since the M205R mutation introduces a charged amino acid, we reasoned that it might be possible to isolate genetic suppressors of this mutation that restore Tat transport activity. Such suppressors could potentially fall within any of the Tat components since it is known that TatC interacts with TatB and TatA, as well as itself (Bolhuis *et al*., 2001; Punginelli *et al*., 2007; Fritsch *et al*., 2012). We initially focussed on the TatB partner protein. We constructed a random mutant library covering the transmembrane domain of TatB, because this region of the protein is most likely to interact with the membrane-integral parts of TatC (Maldonado *et al*., 2011b). To this end, the region covering *tatB* codons 2 to 20 from plasmid pTAT101 C-M205R encoding the TatC M205R mutation was amplified by error prone PCR to serve as a primer in a subsequent site-directed mutagenesis reaction of the same plasmid. This yielded a *tatB* mutant library of around 40 000 individual clones with an average error rate of 4% (2.5 ± 1.5 errors per *tatB*; determined by sequencing 20 random clones).

**Fig. 7:**
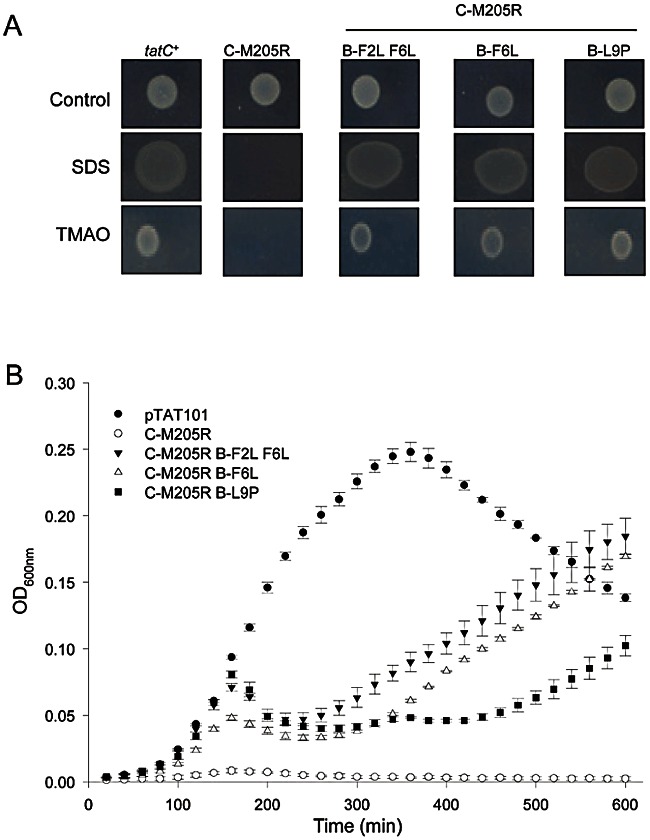
The transport-inactive TatC M205R mutation can be suppressed by mutations in the TatB transmembrane helix. A. Spot tests of strain DADE (Δ*tatABCD*, Δ*tatE*) harbouring a plasmid vector encoding wild-type TatA TatB and TatC (*tatC*^+^), or the same plasmid encoding TatA, the TatC M205 variant and either wild-type TatB (designated C-M205R) or the TatB variants F2L/F6L, F6L or L9P, as indicated. Strains were spotted onto LB medium (Control) or LB medium containing 2% SDS and incubated aerobically overnight. Strains were also spotted onto M9 medium containing glycerol and TMAO and incubated for 3 days under anaerobic conditions. B. Growth curves of the same strain and plasmid combinations in LB liquid medium containing 0.5% SDS. Growth curves were recorded for 10 h in 96-well plates (200 µl volume) without shaking. The average and standard deviation of three replicates are shown.

This library was subsequently screened following transformation into *E. coli* strain DADE (lacking all chromosomally encoded *tat* genes) and selection for aerobic growth on SDS and anaerobic growth on TMAO. An estimated 370 000 colonies were screened, giving in excess of ninefold coverage of the library, and 14 mutants were isolated that conferred the ability to grow on SDS and TMAO. Five different classes of mutations in *tatB* were identified from the 14 clones by sequencing the *tatB* gene, and the whole *tatABC* operon of at least one representative from each class was sequenced to ensure that the TatC M205R substitution was still present and that no further mutations were present in any of the *tat* genes that might compensate for the M205R mutation (Table 4). We confirmed three different TatB mutations that could suppress the inactive TatC M205R phenotype – either of F6L or L9P single amino acid changes or an F2L/F6L double substitution (Fig. 7). Each of the suppressor mutations restored a wild-type behaviour in phenotypic screens on solid media (Fig. 7A), but growth in liquid cultures supplemented with SDS revealed that the suppression was only partial (Fig. 7B). The F6L substitution appeared more effective at suppressing the inactive TatC M205R mutation than the L9P substitution, and the F2L/F6L double substitution was slightly more effective than the F6L substitution alone.

**Table 4:** Amino acid sequence changes identified in clones that suppressed the Tat transport inactivity of a plasmid carrying the TatC M205R mutation

Mutant class	# of times isolated	TatB mutation	TatC mutation
1	3	F2L F6L	M205R
2	1	F6L	M205R
3	8	L9P	M205R
4	1	G5D F13S	M205 wt
5	1	V12A L17F L20P P21T	M205S

### A site-specific contact between TatC transmembrane helix 5 and the transmembrane helix of TatB

**Fig. 8:**
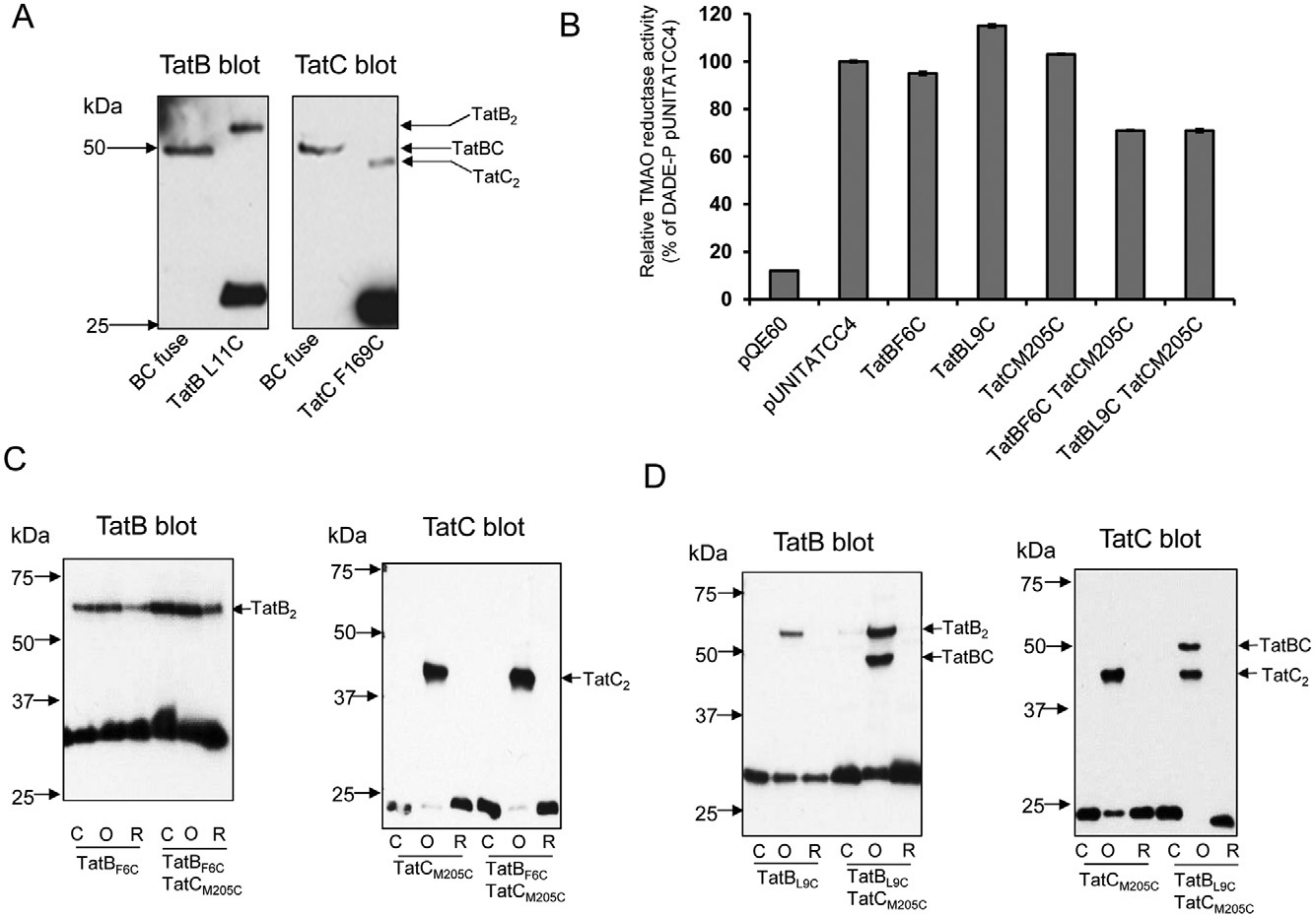
The TatC M205C variant forms a disulphide cross-link with TatB L9C. A. Western blot analysis of a plasmid-produced TatBC fusion protein and comparison with the migration of TatB and TatC cross-linked homodimers. Membrane fractions were prepared from strain DADE-P producing the TatB L11C variant (from plasmid pUNIPLB11; left panel, second lane), the TatC F169C variant (from plasmid pUNICPC169; right panel, second lane), or the TatBC fusion protein (from plasmid pUNITAT1-BCfuse; left and right panel, first lane). Note that the TatB and TatC variants used here were chosen for calibration. Membrane fractions containing TatB L11C and TatC F169C were oxidized for 1 h to generate cross-linked homodimers prior to SDS-PAGE. The blot on the left is incubated with anti-TatB and on the right with anti-TatC antibodies. B. Cysteine-substituted TatB and TatC proteins support Tat transport activity. TMAO reductase activities were measured from the periplasmic fractions of strain DADE-P carrying pQE60 (labelled empty vector), or the same vector producing TatA, TatB and TatC (which has ala substitutions of all four native cys residues), with the indicated cysteine substitutions in TatB and/or TatC. The error bars represent standard error of the mean (*n* = 3). C and D. Membrane samples were prepared from the strain DADE-P co-producing the single cysteine-substituted TatB variants F6C (C) or L9C (D) together with TatA and TatC M205C. Samples (100 µg of membrane protein) were subjected to either oxidizing (labelled O) or reducing conditions (labelled R) or incubated with buffer alone (labelled C). Samples (10 µg of membrane protein) were resolved by SDS-PAGE (10% acrylamide), and proteins were visualized by immunoblotting using anti-TatB (left panels) or anti-TatC antibodies (right panels). TatB and TatC homodimer forms and the TatBC heterodimer are indicated.

The existence of compensatory mutations in the transmembrane domain of TatB that suppress an inactivating mutation in TM5 of TatC suggests that there might be a direct interaction between these regions of the proteins. To probe this further we assessed whether position 205 of TatC, when mutated to a cysteine residue, could form a disulphide cross-link to cysteines introduced into positions 6 or 9 within the transmembrane domain of TatB. To this end we constructed plasmids encoding TatA along with TatC M205C and either wild-type TatB or cysteine variants F6C or L9C of TatB. These cysteine substitutions did not prevent Tat transport activity as judged by the assessment of TMAO reductase activity in the periplasmic fractions of these strains (Fig. 8B). We next prepared membrane fractions from strains expressing each of the single cysteine variants (TatB F6C, TatB L9C or TatC M205C) and the strains co-producing the TatB and TatC cys variants (i.e. TatB F6C or L9C **plus** TatC M205C) and tested them for disulphide-bonded heterodimer formation under oxidizing conditions.

As shown in Fig. 8C and D, producing the TatC M205C substitution along with wild-type TatB gave an anti-TatC-reacting band under oxidizing conditions corresponding to a dimeric form of TatC. Disulphide-bonded homodimers of TatC have been previously detected for the M205C substitution (Punginelli *et al*., 2007). Disulphide-bonded homodimers of TatB were also detected for TatB F6C and L9C when co-produced with cys-less TatC, again as previously reported (Lee *et al*., 2006). When the M205C variant of TatC was co-produced with the F6C variant of TatB, only TatB and TatC homodimers were detected for the oxidized sample (Fig. 8C). However, when the M205C variant of TatC was co-produced with the L9C variant of TatB, a further band appeared when membrane fractions were oxidized (Fig. 8D). This band, which cross-reacted with both the TatB and TatC antibodies, migrated slightly above the TatC homodimer and below the TatB homodimer and co-migrated with a genetically engineered covalent TatB–TatC fusion (Fig. 8A). It thus corresponds to a site-specific TatB–TatC cross-link. Taken together with the results of the genetic suppression analysis these data clearly show that there is a specific contact site between the transmembrane domain of TatB and transmembrane helix 5 of TatC.

## Discussion

In this study we have taken a random mutagenesis approach to isolate mutations that abolish the activity of the TatC protein, with the aim of gaining further insight into its structure and function. Previous directed mutagenesis approaches have highlighted conserved residues in the first cytoplasmic loop as essential for Tat transport activity, possibly by facilitating recognition of the twin arginine signal peptides of Tat substrates (Allen *et al*., 2002; Buchanan *et al*., 2002; Barrett and Robinson, 2005; Holzapfel *et al*., 2007; Strauch and Georgiou, 2007; Zoufaly *et al*., 2012). While our study also identified key residues in this region of the protein, surprisingly the majority of inactivating substitutions were isolated in the first two periplasmic loops of TatC. Despite a lack of notable sequence conservation in these regions, there is conservation of predicted secondary structural elements, principally extensions of the N-terminal ends of the α-helices that lead into transmembrane domains 2 and 4 and the C-terminal end of transmembrane domain 1. Additional insertion and deletion analysis supported the idea that these extended helical regions, particularly in the first periplasmic loop, are essential for Tat transport.

It is not clear what roles the periplasmic regions of TatC might fulfil. The majority of the inactivating mutations we identified in these regions did not grossly affect insertion of TatC into the membrane or induce instability. Furthermore, the four inactivating substitutions in the P1 loop and two in the P2 loop that we tested still assembled into TatBC complexes of a similar size and composition to wild type. It has been reported that the P48A substitution of TatC, which inactivates TatC function, destabilizes the TatBC complex (Barrett *et al*., 2005). By contrast the inactivating P48S substitution we isolated here did not obviously affect complex stability. A recent site-specific photo-cross-linking study where the Bpa cross-linker replaced amino acid L49 failed to give any cross-links with TatB, nor did the same cross-linker situated in the same loop, at residue 63 (Zoufaly *et al*., 2012). This would be consistent with the suggestion that the P1 loop does not form a primary contact site with TatB. Substitution of the Bpa cross-linker at position 63 inactivated the function of TatC, which in agreement with our findings that the D63V substitution also inactivated TatC. It was also shown that TatC self-adducts were formed upon UV irradiation, placing residue 63 in close proximity to a neighbouring TatC (Zoufaly *et al*., 2012).

The presence of an aspartic acid residue (D150) in the P2 loop, just prior to the start of the α-helical region leading into transmembrane domain 4 appears to be particularly important, since we identified two inactivating substitutions of this residue. Likewise, a Bpa residue at this position of TatC also completely inactivated the protein, and interestingly gave cross-linked adducts to both TatB and TatA (Zoufaly *et al*., 2012). A previous disulphide mapping study identified G144C and S148C substitutions in the P2 loop as being in close proximity to the same residues in a neighbouring TatC. A G144C substitution in this loop gave almost 100% conversion to disulphide-bonded dimer through cross-linking to a nearby G144C residue, and likewise an S148C also gave a self-cross-link (Punginelli *et al*., 2007). This is consistent with the P2 loop making multiple contacts to Tat components, possibly at different stages during Tat transport.

**Fig. 9:**
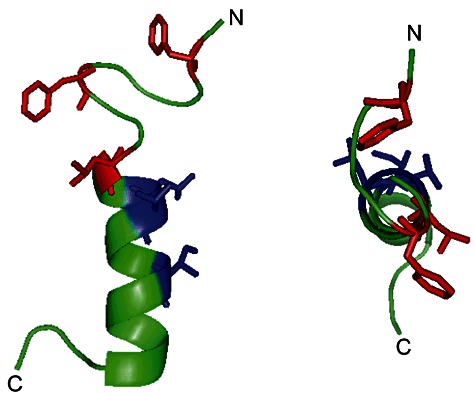
Model of the TatB transmembrane helix. A homology model of *E. coli* TatB was generated using the Swiss-Model server (http://swissmodel.expasy.org; Arnold *et al*., 2006) and the structure of the TatA_d_ protein from *B. subtilis* (Hu *et al*., 2010, PDB code 2l16) as a template. The model predicted the structure of TatB residues 1 to 51, with residues 1 to 23 displayed in the figure. Residues shown in red are the positions of TatB suppressor mutations that restore Tat transport activity synthetically with the TatC M205R substitution. Residues shown in blue are those that are predicted to be involved in TatB-TatB interactions identified by the disulphide cross-linking study of Lee *et al*. (2006). The helix is shown as viewed from the side (*left*) or top (*right*).

Our study also identified three inactivating substitutions that introduced basic amino acids into transmembrane helix 5. One of these substitutions, G204R, clearly affected stability of TatC, while substitutions of M205 to R or K did not prevent TatC from being inserted into the membrane. Interestingly, despite the fact that the M205R substitution did not obviously affect the assembly of the TatBC complex, we were able to suppress this inactivating mutation with compensatory mutations in the transmembrane helix of TatB. This would be consistent with the idea that introduction of an arginine residue at position 205 causes only local structural disruption, and that substitution of one or more bulky residues in the transmembrane helix of TatB might relieve this perturbation by facilitating packing of the arginine. A direct interaction between the transmembrane helix of TatB and transmembrane helix 5 of TatC is consistent with the finding that a cysteine substitution of TatC 205 can form a disulphide bond with a cysteine residue at position 9 of TatB. It is also in agreement with previous findings that the D211A substitution of TatC, which is located at the C-terminal end of transmembrane helix 5, destabilizes the TatBC complex (Buchanan *et al*., 2002). Using the NMR structure of the TatA_d_ protein from *Bacillus subtilis* as a template (Hu *et al*., 2010; PDB code 2l16), we generated a homology model of the transmembrane helix of *E. coli* TatB to visualize the positions of TatB residues involved in TatB–TatB and TatB–TatC interactions (Fig. 9). Residues in TatB are colour-coded according to the different types of interactions, with residues shown in blue on Fig. 9 being those that have been previously identified to give strong TatB self-cross-links when individually substituted to cysteine (Lee *et al*., 2006). The positions of the suppressor mutations that synthetically restore Tat transport activity in concert with the TatC M205R mutation are shown in red. These suppressors map away from the predicted TatB self-interaction interface.

A mutation in the transmembrane domain of TatB has also been shown to confer relaxed signal sequence specificity, such that a signal peptide with a normally inactive KQ substitution of the conserved twin arginine motif also directed Tat transport (Kreutzenbeck *et al*., 2007). This mutation, which substitutes glutamate at position 8 with lysine, is next to the position 9 suppressor of the TatC M205R mutation. It is unlikely that this region of TatB directly contacts the twin arginine motif because site-specific photo cross-linking indicates that this region of the signal peptide interacts predominantly with TatC, whereas TatB forms prominent cross-links with the signal peptide h-region (Alami *et al*., 2003). It has been proposed that the E8K substitution of TatB mediates long range conformational effects altering the signal peptide binding pocket (Kreutzenbeck *et al*., 2007). It is reasonable to suppose that these changes might be mediated through modulation of the interaction of TatB with transmembrane helix 5 of TatC.

While it has been known for some time that TatB and TatC form a stable complex, molecular details on specific sites of contact between the two proteins has been lacking (e.g. Bolhuis *et al*., 2001; Maldonado *et al*., 2011b). In this study we have reported the first TatB-C site-specific contact. It is interesting to note that both proteins also show some degree of self-cross-linking as well as the formation of an interspecies disulphide bond. This may be because the introduced cysteine residues are at interface sites, so that both the same and different types of subunit are accessible for cross-linking. Alternatively it may reflect conformational or positional changes in TatB and TatC during the Tat transport cycle such that homo-dimeric cross-linking occurs in one part of the cycle and hetero-dimeric in another. It will be interesting to see whether co-overproduction of a substrate protein and/or the absence of TatA/E influence the degree of homo-dimeric versus hetero-dimeric cross-linking. Furthermore, it has recently been shown that when TatB is absent, a complex containing *E. coli* TatA and TatC can be isolated (Fritsch *et al*., 2012). If TatA occupies the same binding site on TatC as TatB does, then it is possible that similar site-specific cross-links might be detected between cysteine residues in transmembrane helix 5 of TatC and TatA.

## Experimental procedures

### Bacterial strains and plasmids

Strains MC4100 (F^-^, *[araD139]_B/r_*, Δ(*argF-lac*)*U169*, *λ*^-^, *e14*-, *flhD5301*, Δ(*fruK-yeiR*)*725*(*fruA25*), *relA1*, *rpsL150*(Str^R^), *rbsR22*, Δ(*fimB-fimE*)*632*(*::IS1*), *deoC1*; Casadaban and Cohen, 1979) and DADE (as MC4100, Δ*tatABCD*, Δ*tatE*; Wexler *et al*., 2000) were used in this study. Plasmid pTAT1d contains a modified *tatABC* operon expressed from the native *tatA* promoter in plasmid pT7.5 and has been described previously (Maldonado *et al*., 2011b). Plasmid pTAT101 was constructed by excision of the *tat* promoter and the *tatABC* operon as an *Eco*RI/*Pst*I fragment and subcloning into the low copy number plasmid pTH19kr (Hashimoto-Gotoh *et al*., 2000). For TatBC complex isolation, his-tagged TatC variants were co-produced with TatA and TatB from plasmid pUNITAT2 (McDevitt *et al*., 2005). For disulphide cross-linking experiments, TatB and TatC variants were co-produced with TatA from plasmid pUNITATCC4 (Lee *et al*., 2006). To make a construct that produces a TatBC fusion protein, the strategy outlined by Bolhuis *et al*. (2001) was followed exactly. DNA covering the *tatB-C* fusion was subcloned into pUNITAT1 (Lee *et al*., 2006) to give plasmid pUNITAT1-BCfuse. A complete list of all plasmids used in this study is given in Table S2.

### PCR mutagenesis and library construction

For random PCR mutagenesis of *tatC*, plasmid pTAT1d (Maldonado *et al*., 2011b) was used as template. The region encompassing *tatC* was amplified in the presence of MnCl_2_ (Fromant *et al*., 1995) using flanking primers tatCm6 and tatCm7 (Table S3). PCR conditions were adjusted to result in an average error rate of approximately 0.2%. PCR products were purified with the Qiaquick PCR purification kit (Qiagen), restricted with *Xho*I and *Pst*I and cloned back into pTAT1d to replace native *tatC*. Recombinant plasmids were used to transform *E. coli* XL10-Gold cells (Tet^r^Δ(*mcrA*)*183*Δ(*mcrCB-hsdSMR-mrr*)*173 endA1 supE44 thi-1 recA1 gyrA96 relA1 lac* Hte [F' *proAB lacI*^q^*Z*Δ*M15* Tn*10* (Tet^r^) Amy Cam^r^]– Stratagene). Twenty random colonies were picked and the *tatC* gene sequenced to evaluate the overall error rate. Remaining colonies (approximately 600 000) were resuspended in LB and used to inoculate 200 ml of LB supplemented with ampicillin to give a starting OD_600_ of 0.2. Cells were grown aerobically until a final OD_600_ of around 2 was reached and plasmid DNA was isolated from 20% of the culture volume and stocked as the *tatC* mutant library. The *tatC* library was screened for clones that failed to export an artificial substrate in which the CAT has been fused to the TorA signal peptide according to a procedure described previously (Maldonado *et al*., 2011b).

Random mutagenesis of DNA encoding the TatB transmembrane region was carried out according to an alternative PCR mutagenesis procedure (Vartanian *et al*., 1996). A 57 bp fragment comprising *tatB* codons 2 to 20 inclusive was amplified using primers TatBtmsupp1 and TatBtmsupp2 (Table S3) in the presence of 0.5 mM MnCl_2_ and strong dNTP bias (dTTP and dGTP at a concentration of 1000 µM each versus dATP and dCTP at 80 µM). The resulting PCR product was purified as described above and used as a primer in a subsequent site-directed mutagenesis step according to the Quickchange protocol (Stratagene). PCR products were digested with *Dpn*I, ethanol precipitated and used to transform XL10-Gold cells. Twenty random colonies were picked and the *tatB* gene sequenced to evaluate the overall error rate, and the remaining colonies (approximately 40 000) were used to prepare a library as described above.

To introduce nucleotide exchanges as well as insertions and deletions into *tatC* the Quickchange site-directed mutagenesis protocol (Stratagene) was followed. All primers used for site-directed mutagenesis are listed in Table S3. Amplified products were digested with *Dpn*I and subsequently used to transform *E. coli* strain DH5α (*F*^-^ϕ*80dlacZM15* (*lacZYA-argF*)*U169 deoR recA1 endA1 hsdR17*(*r k*^-^*, mk*^+^*) phoA supE44 thi-1 gyrA96 relA1*λ^-^). Modified plasmids were isolated and the introduction of the selected mutations was confirmed by sequencing of the whole *tatABC* operon.

### Phenotypic assessment of Tat transport activity

The growth of *E. coli* strain DADE producing plasmid encoded TatA, TatB and variant TatC proteins was assayed aerobically in the presence of SDS and anaerobically with TMAO as described previously (Buchanan *et al*., 2002; Palmer *et al*., 2010). Strains that were judged to be devoid of Tat activity on the basis of these tests were further tested for growth in liquid media containing SDS in 96-well format. Two hundred microlitres aliquots of LB medium containing kanamycin and 0.5% (w/v) SDS were inoculated with an overnight culture at 1:100 dilution. Cells were grown without shaking for 8 to 10 h in a Synergy 2 microplate reader (Biotek) at 37°C and the OD_600_ recorded.

For measurement of TMAO reductase activity, strains were grown anaerobically overnight at 37°C in LB containing 0.4% (w/v) TMAO and 0.5% (v/v) glycerol. Periplasmic fractions were prepared using EDTA/lysozyme treatment and TMAO:benzyl viologen oxidoreductase activity in the periplasmic fraction was measured as described previously (Silvestro *et al*., 1988; Palmer *et al*., 2010).

### Membrane protein preparation

*Escherichia coli* membrane fractions for TatC immunoblotting were prepared from 25 ml LB cultures that had been inoculated with a 1:100 dilution of an overnight culture of the strain of interest and grown aerobically for 6 h. Cells were harvested by centrifugation and resuspended in 1 ml 50 mM Tris-HCl, pH 7.6, 5 M Urea to wash off membrane-associated proteins during membrane preparation. Cells were broken by sonification and cell debris removed by centrifugation at 16 000 *g* for 10 min. The supernatant was subjected to ultracentrifugation at 227 000 *g* for 30 min and the resulting membrane pellet resuspended in 70 µl 50 mM Tris-HCl, pH 7.6. Total membrane protein was quantified using the DC Protein Assay kit (Bio-Rad). For TatC immunoblotting 40 µg of total membrane protein was separated by SDS-PAGE (12% acrylamide) (Laemmli, 1970) and transferred to PVDF membranes (Towbin *et al*., 1979). Immunoreactive bands were visualized using an anti-TatC antibody (Alami *et al*., 2002) and the Immobilon Western kit (Milipore).

For the purification of TatBChis complexes cells were grown at 37°C in 50 ml LB medium until an OD_600_ of 0.7 was reached, at which point expression of the plasmid-borne *tat* genes was induced by the addition of 0.5 mM IPTG. The cells were then grown for another 3 h before being harvested by centrifugation (3000 *g*, 15 min), resuspended in 20 ml of buffer (20 mM MOPS pH 8, 200 mM NaCl), and lysed by one French Press passage at 12 000 psi. The lysate supernatant was cleared from cellular debris by 10 min centrifugation at 12 000 *g*, and the membrane fraction was sedimented by centrifugation at 150 000 *g* for 1 h. The pelleted membranes were resuspended in 1.5 ml of buffer and solubilized by adding 1.5 ml of buffer containing 2% digitonin and incubating for 2 h at RT. The supernatant was then cleared by centrifugation at 120 000 *g* for 30 min. To isolate hexahistidine-tagged proteins, 750 µl of supernatant was incubated with 50 µl HisMag (Promega) beads for 15 min. The beads were washed five times with 200 µl of buffer containing 0.15% digitonin and 50 mM imidazole before elution with 50 µl of the same buffer containing 500 mM imidazole. BN-PAGE was performed using precast gels (NativePAGE, Invitrogen) as per the manufacturer's instructions, and SDS-PAGE of purified TatBC samples utilized 4–20% precast gels (TGX, Bio-Rad). Western blotting was carried out using PVDF membranes (iBlotter, Invitrogen). After electroblotting, TatB and TatC were identified using polyclonal antisera to each protein raised in rabbits (Sargent *et al*., 2001; Alami *et al*., 2002).

### Disulphide cross-linking

Disulphide cross-linking experiments were performed from a modified protocol based on (Lee *et al*., 2006). *E. coli* strain DADE-P, producing plasmid-encoded cysteine-substituted variants of TatB and/or TatC (along with wild-type TatA) was grown overnight at 37°C in 50 ml LB cultures without the addition of IPTG. Cells were harvested, washed and resuspended with 1 ml of Buffer A (20 mM Tris-HCl, pH 7.5, 200 mM NaCl) supplemented with a protease inhibitor cocktail (Roche). Cells were disrupted by sonication and cell debris was removed by a low speed centrifugation step. The supernatant was further ultra-centrifuged (200 000 *g* 30 min, 4°C) and the membrane pellet was resuspended in 100 µl of Buffer B (50 mM Tris-HCl, pH 7.5, 5 mM MgCl_2_, 10% glycerol). Disulphide cross-linking experiments were carried out with membranes (100 µg of total protein) at room temperature for 1 h. Oxidized, reduced, and control samples were run simultaneously. A final concentration of 2 mM copper phenanthroline was used in the oxidized sample and after 1 h incubation the reaction was quenched for 10 min at room temperature by the addition of 25 mM *N*-ethylmaleimide, 50 mM Na_2_EDTA, pH 7.5 (final concentration). Reduced samples were incubated for 1 h with 10 mM dithiothreitol, while the control sample was incubated for 1 h with Buffer B alone. After incubation all samples was made up to 100 µl with Laemmli buffer prior to analysis by SDS-PAGE (10% acrylamide) and Western blotting using nitrocellulose membrane (GE-Healthcare).
