# Supplementary material for: Molecular dissection of TatC defines critical regions essential for protein transport and a TatB–TatC contact site
Source: Mol Microbiol. 2012 Jul 13;85(5):945–61. doi: 10.1111/j.1365-2958.2012.08151.x (PMC3712464; doi:10.1111/j.1365-2958.2012.08151.x)
Supplement: Supplementary file 1 [file mmi0085-0945-SD1.pdf]

Figure S1. Schematic representation of the *tatABC* region of plasmid pTAT1d. The nucleotide sequence around the start and stop codons of the *tatABC* genes is shown, with the amino acid sequence beneath. Introduced restriction sites are underlined with nucleotides differing from the original sequence shown in bold and resulting amino acid changes italicised. The *tatC* mutant library was cloned as a *Xho*I - *Pst*I fragment. epPCR – error prone PCR. Restriction sites are GGATCC – *Bam*HI; AGATCT – *Bgl*II; CACGTG – *Pml*I; CTCGAG – *Xho*I; CTTAAG – *Afl*II; GTCGAC – *Sal*I; CTGCAG – *Pst*I.

Figure S2. Production of TatC from plasmid pTAT101. A. Total membrane fractions were prepared from strain MC4100 (*tat*$^+$) and DADE (Δ*tatABCD*, Δ*tatE*) harbouring plasmid pTAT101 which encodes *tatABC*. The indicated amounts of total membrane protein were subjected to SDS-PAGE and subsequent immunoblotting using an anti-TatC antibody. B. The intensity of each band following Western blotting was quantified using ImageJ.

Figure S3. Alignment of selected bacterial TatC proteins. Protein sequences were aligned using jpred (Cole *et al.*, 2008); with predicted cytoplasmic residues shaded green, transmembrane in red and periplasmic in purple. Eco - *Escherichia coli*, Cje -*Campylobacter jejuni*, Dvu - *Desulfovibrio vulgaris*, Son - *Shewanella oneidensis*, Psy- *Pseudomonas syringae* pv tomato, Xor - *Xanthomonas oryzae* pv oryzae, Lph - *Legionella pneumophila* subsp *pneumophila*, Dda - *Dickeya dadantii*, Bsu_C_d- *Bacillus subtilis* TatCd, Bsu_Cy - *Bacillus subtilis* TatC_y, Syn - *Synechocystis* sp, Sau - *Staphylococcus aureus*, Sli - *Streptomyces lividans*, Atu - *Agrobacterium tumefaciens*, Mxa - *Myxococcus xanthus*, Yps - *Yersinia pseudotuberculosis*, Msm - *Mycobacterium smegmatis*.

clone	TatC mutation/s
103	V64A
104	S79L G144R E257G
105	P48S A133V
110	F94S Y154C
111	R17C I60N D188V
113	L16Q L74Q L82P P209S T216A
117	L16P L53S
123	Q215R
124	Q52L G144E Q146R S153I
126	M205K S214P
128	L20P Y154H
130	C33S S66P L74P K192E
132	I25N F31I I60F F68S P131T aa251-258 AESEKTEE→QKRKTEE
133	M59K V198D
138	D63V
139	P67S P131S I183T
201	A47S F68L I175F Y223C Stop TAA→TAT 14 aa C-terminal extension YGCSPSLSMISCQT
205	L9H C23R A26V S57P G199S
207	Q52L G144E Q146R S153I
209	V35D F68S S115Y I175V L177P L178P F213S E252G
212	L116P F124S V176E L217P
214	S66P A98V
224	V64E F232S
225	L137H A141E F232L
226	G204R frameshift aa 241 altering C-terminus

303	F157Y
311	A160T
318	A160V A219E
321	V3A P67S E103K
323 [a]	M163K M205K
328 [a]	P48L
329	H12R P109A A160V
333	L34P H43Y A25T T208A
335 [a]	S148P
336 [a]	L137H S214P
341 [a]	L20P L111P E143G
344	D63V Y100C
351	R19C P54L R105C
354 [a]	F37S Q90R F94L
360	L49S E103G P210T
366 [a]	N39S P54L
367 [a]	C23R
368	F68S
375	Y42N L99P
403	H12R R17H
407	R19C P54L F130S P209L A219S
409	E103G G229D
411	H12R I87T S168P T216S
415	L16P
434	G121D M205R
437 [a]	L99P
440	L116R L155V

441	I72F V145E P209L G238D
445	P54S D150G T184S
446	L74P Y89C S113I F130I
447	Y42C S46F D150V 14aa N-terminal extension as 201
451	E15G I151T
457	P97T V203I L225P
470	T62A M222I
475	L9H A65T P71A S79L L99Q D150N D201N E250V
479	C23R I41N L206M M222L
482	M59K
485	C23R N139K E227K
490	R19S F94I Q146K F149I
495	L99Q L178Q
496	C33Y A38T H43Y S57L S158P W180R D248G
497	S148P V168A
510 [a]	E15G E103G F127I L217P
513	L82Q P85L
519	P48S
521	P8S A47T Y100H L111P
523	L59S Q52L F94L
533 [a]	S66P L106P
544	L20P T70R A160T
546	R104C P131L C224S
549	A61T S148P E252Stop
556	E15K R17S F165L
557	S83T Y100C P210R
558	L20P I60V F130L

Table S1. Amino acid sequence changes found in clones isolated from the *tatC* mutant library giving rise to an inactive Tat system. Note that clones containing frame shifts in the essential or stop codons in the region of TatC up to the end of transmembrane helix 6 are not included.

Plasmid	Description	Reference
pTH19kr	Low copy-number cloning vector (kan^r)	Hashimoto-Gotoh *et al*. (2000)
pTAT1	pUNI-PROM carrying *E. coli tatABC* from pUNITAT1	This work
pTAT1d	pUNI-PROM carrying *E. coli tatABC* with engineered restriction sites allowing facile replacement of each gene	Maldonado *et al*. (2011b)
pTTC1	pSU40 encoding TorA$_{ss}$-CAT fusion	Maldonado *et al*. (2011b)
pTAT101	pTH19kr carrying *tat* promoter and *tatABC* fragment from pTAT1	This work
pQE60	Expression vector (amp^r)	Qiagen
pUNITAT1	*tatABC* operon in pQE60	Lee *et al*. (2006)
pUNITATCC4	*tatABC* operon in pQE60, all 4 cysteine codons in *tatC* substituted for alanine codons	Lee *et al*. (2006)
pUNITAT2	*tatABC* operon in pQE60, *tatC* fused to 3' hexahistidine coding sequence.	McDevitt *et al*. (2005)
pTAT101 C-E15G	As pTAT101 but TatC E15G exchange	This work
pTAT101 C-L16P	As pTAT101 but TatC L16P exchange	This work
pTAT101 C-R17H	As pTAT101 but TatC R17H exchange	This work
pTAT101 C-L20P	As pTAT101 but TatC L20P exchange	This work
pTAT101 C-C23R	As pTAT101 but TatC C23R exchange	This work
pTAT101 C-L34P	As pTAT101 but TatC L34P exchange	This work
pTAT101 C-Y42N	As pTAT101 but TatC Y42N exchange	This work
pTAT101 C-S46F	As pTAT101 but TatC S46F exchange	This work
pTAT101 C-A47T	As pTAT101 but TatC A47T exchange	This work
pTAT101 C-P48L	As pTAT101 but TatC P48L exchange	This work
pTAT101 C-P48S	As pTAT101 but TatC P48S exchange	This work
pTAT101 C-L49S	As pTAT101 but TatC L49S exchange	This work
pTAT101 C-L53P	As pTAT101 but TatC L53P exchange	This work
pTAT101 C-L53S	As pTAT101 but TatC L53S exchange	This work
pTAT101 C-P54L	As pTAT101 but TatC P54L exchange	This work
pTAT101 C-M59K	As pTAT101 but TatC M59K exchange	This work
pTAT101 C-I60N	As pTAT101 but TatC I60N exchange	This work
pTAT101 C-A61T	As pTAT101 but TatC A61T exchange	This work

pTAT101 C-A61V	As pTAT101 but TatC A61V exchange	This work
pTAT101 C-T62A	As pTAT101 but TatC T62A exchange	This work
pTAT101 C-D63V	As pTAT101 but TatC D63V exchange	This work
pTAT101 C-V64A	As pTAT101 but TatC V64A exchange	This work
pTAT101 C-V64D	As pTAT101 but TatC V64D exchange	This work
pTAT101 C-V64E	As pTAT101 but TatC V64E exchange	This work
pTAT101 C-S66P	As pTAT101 but TatC S66P exchange	This work
pTAT101 C-P67S	As pTAT101 but TatC P67S exchange	This work
pTAT101 C-T70R	As pTAT101 but TatC T70R exchange	This work
pTAT101 C-L74P	As pTAT101 but TatC L74P exchange	This work
pTAT101 C-S79L	As pTAT101 but TatC S79L exchange	This work
pTAT101 C-L82Q	As pTAT101 but TatC L82Q exchange	This work
pTAT101 C-P85L	As pTAT101 but TatC P85L exchange	This work
pTAT101 C-Q90R	As pTAT101 but TatC Q90R exchange	This work
pTAT101 C-F94L	As pTAT101 but TatC F94L exchange	This work
pTAT101 C-F94S	As pTAT101 but TatC F94S exchange	This work
pTAT101 C-P97T	As pTAT101 but TatC P97T exchange	This work
pTAT101 C-A98V	As pTAT101 but TatC A98V exchange	This work
pTAT101 C-L99P	As pTAT101 but TatC L99P exchange	This work
pTAT101 C-L99Q	As pTAT101 but TatC L99Q exchange	This work
pTAT101 C-Y100C	As pTAT101 but TatC Y100C exchange	This work
pTAT101 C-E103G	As pTAT101 but TatC E103G exchange	This work
pTAT101 C-E103K	As pTAT101 but TatC E103K exchange	This work
pTAT101 C-R104C	As pTAT101 but TatC R104C exchange	This work
pTAT101 C-R105C	As pTAT101 but TatC R105C exchange	This work
pTAT101 C-P109A	As pTAT101 but TatC P109A exchange	This work
pTAT101 C-L111P	As pTAT101 but TatC L111P exchange	This work
pTAT101 C-L116P	As pTAT101 but TatC L116P exchange	This work
pTAT101 C-L116R	As pTAT101 but TatC L116R exchange	This work
pTAT101 C-G121D	As pTAT101 but TatC G121D exchange	This work
pTAT101 C-F130S	As pTAT101 but TatC F130S exchange	This work
pTAT101 C-P131L	As pTAT101 but TatC P131L exchange	This work
pTAT101 C-A133V	As pTAT101 but TatC A133V exchange	This work
pTAT101 C-L137H	As pTAT101 but TatC L137H exchange	This work
pTAT101 C-A141E	As pTAT101 but TatC A141E exchange	This work
pTAT101 C-E143G	As pTAT101 but TatC E143G exchange	This work

pTAT101 C-G144R	As pTAT101 but TatC G144R exchange	This work
pTAT101 C-V145E	As pTAT101 but TatC V145E exchange	This work
pTAT101 C-V147E	As pTAT101 but TatC V147E exchange	This work
pTAT101 C-S148P	As pTAT101 but TatC S148P exchange	This work
pTAT101 C-D150G	As pTAT101 but TatC D150G exchange	This work
pTAT101 C-D150V	As pTAT101 but TatC D150V exchange	This work
pTAT101 C-I151T	As pTAT101 but TatC I151T exchange	This work
pTAT101 C-Y154H	As pTAT101 but TatC Y154H exchange	This work
pTAT101 C-F157Y	As pTAT101 but TatC F157Y exchange	This work
pTAT101 C-A160V	As pTAT101 but TatC A160V exchange	This work
pTAT101 C-M163K	As pTAT101 but TatC M163K exchange	This work
pTAT101 C-S168P	As pTAT101 but TatC S168P exchange	This work
pTAT101 C-D188V	As pTAT101 but TatC D188V exchange	This work
pTAT101 C-V198D	As pTAT101 but TatC V198D exchange	This work
pTAT101 C-G204R	As pTAT101 but TatC G204R exchange	This work
pTAT101 C-M205K	As pTAT101 but TatC M205K exchange	This work
pTAT101 C-M205R	As pTAT101 but TatC M205R exchange	This work
pTAT101 C-P209L	As pTAT101 but TatC P209L exchange	This work
pTAT101 C-P210R	As pTAT101 but TatC P210R exchange	This work
pTAT101 C-S214P	As pTAT101 but TatC S214P exchange	This work
pTAT101 C-Q215R	As pTAT101 but TatC Q215R exchange	This work
pTAT101 C-A219E	As pTAT101 but TatC A219E exchange	This work
pTAT101 C-L225P	As pTAT101 but TatC L225P exchange	This work
pTAT101 C-ΔY42	As pTAT101 but TatC Y42 deleted	This work
pTAT101 C-ΔA47	As pTAT101 but TatC A47 deleted	This work
pTAT101 C-ΔK51	As pTAT101 but TatC K51 deleted	This work
pTAT101 C-ΔP54	As pTAT101 but TatC P54 deleted	This work
pTAT101 C-ΔG56	As pTAT101 but TatC G56 deleted	This work
pTAT101 C-ΔT58	As pTAT101 but TatC T58 deleted	This work
pTAT101 C-ΔA61	As pTAT101 but TatC A61 deleted	This work
pTAT101 C-ΔT62	As pTAT101 but TatC T62 deleted	This work
pTAT101 C-ΔA65	As pTAT101 but TatC A65 deleted	This work
pTAT101 C-ΔP67	As pTAT101 but TatC P67 deleted	This work
pTAT101 C-ΔT70	As pTAT101 but TatC T70 deleted	This work
pTAT101 C-ΔP71	As pTAT101 but TatC P71 deleted	This work
pTAT101 C-ΔK73	As pTAT101 but TatC K73 deleted	This work

pTAT101 C-ΔQ55-G56	As pTAT101 but TatC Q55-G56 deleted	This work
pTAT101 C-ΔG56-S57	As pTAT101 but TatC G56-S57 deleted	This work
pTAT101 C-iA42	As pTAT101 but TatC A42 insertion	This work
pTAT101 C-iA47	As pTAT101 but TatC A47 insertion	This work
pTAT101 C-iA51	As pTAT101 but TatC A51 insertion	This work
pTAT101 C-iA54	As pTAT101 but TatC A54 insertion	This work
pTAT101 C-iG56	As pTAT101 but TatC G56 insertion	This work
pTAT101 C-iGG56	As pTAT101 but TatC GG56 insertion	This work
pTAT101 C-iA58	As pTAT101 but TatC A58 insertion	This work
pTAT101 C-iA61	As pTAT101 but TatC A61 insertion	This work
pTAT101 C-iA65	As pTAT101 but TatC A65 insertion	This work
pTAT101 C-iA67	As pTAT101 but TatC A67 insertion	This work
pTAT101 C-iA70	As pTAT101 but TatC A70 insertion	This work
pTAT101 C-ΔA133	As pTAT101 but TatC A133 deleted	This work
pTAT101 C-ΔN139	As pTAT101 but TatC N139 deleted	This work
pTAT101 C-ΔP142	As pTAT101 but TatC P142 deleted	This work
pTAT101 C-ΔQ146	As pTAT101 but TatC Q146 deleted	This work
pTAT101 C-ΔA152	As pTAT101 but TatC A152 deleted	This work
pTAT101 C-ΔA160	As pTAT101 but TatC A160 deleted	This work
pTAT101 C-iA133	As pTAT101 but TatC A133 insertion	This work
pTAT101 C-iA139	As pTAT101 but TatC A139 insertion	This work
pTAT101 C-iA142	As pTAT101 but TatC A142 insertion	This work
pTAT101 C-iA146	As pTAT101 but TatC A146 insertion	This work
pTAT101 C-iA152	As pTAT101 but TatC A152 insertion	This work
pTAT101 C-iA160	As pTAT101 but TatC A160 insertion	This work
pTAT101 C-M205R B-F2L F6L	As pTAT101 C-M205R but TatB F2L F6L exchanges	This work
pTAT101 C-M205R B-F6L	As pTAT101 C-M205R but TatB F6L exchange	This work
pTAT101 C-M205R B-L9P	As pTAT101 C-M205R but TatB L9P exchange	This work
pUNIPLB6	As pUNITATCC4 but TatB F6C exchange	Lee *et al*. (2006)
pUNIPLB9	As pUNITATCC4 but TatB L9C exchange	Lee *et al*. (2006)
pUNIPLB11	As pUNITATCC4 but TatB L11C exchange	Lee *et al*. (2006)
pUNICPC169	As pUNITATCC4 but TatC F169C exchange	Punginelli *et al*. (2007)

pUNICPC205	As pUNITATCC4 but TatC M205C exchange	Punginelli *et al*. (2007)
pUNIC M205C TatBF6C	As pUNICPC205 but additional TatB F6C exchange	This work
pUNIC M205C TatBL9C	As pUNICPC205 but additional TatB L9C exchange	This work
pUNITAT1-BCfuse	As pUNITAT1, but with *tatB* and *tatC* genes fused together with an *Xba*I site. *tatB* stop codon and *tatC* start codon missing.	This work
pUNITAT2 TatC P48S	As pUNITAT2 but TatC P48S exchange	This work
pUNITAT2 TatC I60N	As pUNITAT2 but TatC I60N exchange	This work
pUNITAT2 TatC V64E	As pUNITAT2 but TatC V64E exchange	This work
pUNITAT2 TatC F68S	As pUNITAT2 but TatC F68S exchange	This work
pUNITAT2 TatC L137H	As pUNITAT2 but TatC L137H exchange	This work
pUNITAT2 TatC I151T	As pUNITAT2 but TatC I151T exchange	This work
pUNITAT2 TatC M205R	As pUNITAT2 but TatC M205R exchange	This work

[a] mutants isolated multiple times

Table S2. Plasmids used and constructed in this study.

Primer	Sequence
TatBtmsupp1	CGATAAAGAGCAGGTGTAACACGTG
TatBtmsupp2	ACAGGCAGTCGTTGCGGCCC
tatCm6	CTTCCTCGAGTGATAAACCTTAAGCATG
tatCm7	CCAAGCTTGCATGCCTGCAG
C-E15Gf	CGCATCTGATTGGGCTGCGTAAGCG
C-E15Gr	CGCTTACGCAGCCCAATCAGATGCG
C-L16Pf	CTGATTGAGCCGCGTAAGCGTC
C-L16Pr	GACGCTTACGCGGCTCAATCAG
C-R17Hf	CTGATTGAGCTGCATAAGCGTCTGC
C-R17Hr	GCAGACGCTTATGCAGCTCAATCAG
C-L20Pf	CGTAAGCGTCCGCTGAACTGC
C-L20Pr	GCAGTTCAGCGGACGCTTACG
C-C23Rf	CTGCTGAACCGCATTATCGCGG
C-C23Rr	CCGCGATAATGCGGTTCAGCAG
C-L34Pf	GATATTCCTGTGTCCGGTCTATTTCGC
C-L34Pr	GCGAAATAGACCGGACACAGGAATATC
C-Y42Nf	CCAATGACATCAATCACCTGGTATC
C-Y42Nr	GATACCAGGTGATTGATGTCATTGG
C-S46Ff	CTATCACCTGGTATTCGCGCCATTG
C-S46Fr	CAATGGCGCGAATACCAGGTGATAG
C-A47Tf	CCTGGTATCCACGCCATTGATC
C-A47Tr	GATCAATGGCGTGGATACCAGG
C-P48Lf	GGTATCCGCGCTGTTGATCAAGC
C-P48Lr	GCTTGATCAACAGCGCGGATACC
C-P48Sf	GGTATCCGCGTCATTGATCAAGC
C-P48Sr	GCTTGATCAATGACGCGGATACC

C-L53Pf2	GATCAAGCAGCCGCCGCAAGGTTC
C-L53Pr2	GAACCTTGCGGCGGCTGCTTGATC
C-L53Sf	GATCAAGCAGTCGCCGCAAGGTTC
C-L53Sr	GAACCTTGCGGCGACTGCTTGATC
C-P54Lf	CAAGCAGTTGCTGCAAGGTTCAACG
C-P54Lr	CGTTGAACCTTGCAGCAACTGCTTG
C-M59Kf	GGTTCAACGAAGATCGCCACCG
C-M59Kr	CGGTGGCGATCTTCGTTGAACC
C-I60Nf	GTTCAACGATGAACGCCACCGACG
C-I60Nr	CGTCGGTGGCGTTCATCGTTGAAC
C-A61Tf	CAACGATGATCACCACCGACGTG
C-A61Tr	CACGTCGGTGGTGATCATCGTTG
C-A61Vf	CAACGATGATCGTCACCGACGTG
C-A61Vr	CACGTCGGTGACGATCATCGTTG
C-T62Af	GATGATCGCCGCCGACGTGGCC
C-T62Fr	GGCCACGTCGGCGGCGATCATC
C-D63Vf	GATGATCGCCACCGTCGTGGCCTCGC
C-D63Vr	GCGAGGCCACGACGGTGGCGATCATC
C-V64Af	GCCACCGACGCGGCCTCGCCGTTC
C-V64Ar	GAACGGCGAGGCCGCGTCGGTGGC
C-V64Df	GCCACCGACGACGCCTCGCCGTTC
C-V64Dr	GAACGGCGAGGCGTCGTCGGTGGC
C-V64Ef2	GCCACCGACGAGGCCTCGCCGTTC
C-V64Er2	GAACGGCGAGGCCTCGTCGGTGGC
C-S66Pf	CGACGTGGCCCCGCCGTTCTTTACG
C-S66Pr	CGTAAAGAACGGCGGGGCCACGTCG
C-F68Sf	GGCCTCGCCGTCCTTTACGCCGATC

C-F68Sr	GATCGGCGTAAAGGACGGCGAGGCC
C-L74Pf	CGCCGATCAAGCCGACCTTTATGG
C-L74Pr	CCATAAAGGTCGGCTTGATCGGCG
C-S79Lf	CCTTTATGGTGTTGCTGATTCTGTC
C-S79Lr	GACAGAATCAGCAACACCATAAAGG
C-L82Qf	GTCGCTGATTCAGTCAGCGCCG
C-L82Qr	CGGCGCTGACTGAATCAGCGAC
C-P85Lf	CTGTCAGCGCTGGTGATTCTC
C-P85Lr	GAGAATCACCAGCGCTGACAG
C-P97Tf	CATTTATCGCCACAGCGCTGTATAAG
C-P97Tr	CTTATACAGCGCTGTGGCGATAAATG
C-A98Vf	CATTTATCGCCCCAGTGCTGTATAAGC
C-A98Vr	GCTTATACAGCACTGGGGCGATAAATG
C-L99Pf	CGCCCCAGCGCCGTATAAGCATGAAC
C-L99Pr	GTTCATGCTTATACGGCGCTGGGGCG
C-L99Qf	GCCCCAGCGCAGTATAAGCATGAAC
C-L99Qr	GTTCATGCTTATACTGCGCTGGGGC
C-Y100Cf	CCAGCGCTGTGTAAGCATGAACG
C-Y100Cr	CGTTCATGCTTACACAGCGCTGG
C-R104Cf	GTATAAGCATGAATGTCGCCTGGTG
C-R104Cr	CACCAGGCGACATTCATGCTTATAC
C-R105Cf	GCATGAACGTTGCCTGGTGGTG
C-R105Cr	CACCACCAGGCAACGTTCATGC
C-P109Af	CTGGTGGTGGCGCTGCTGGTTTCC
C-P109Ar	GGAAACCAGCAGCGCCACCACCAG
C-L111Pf	GTGCCGCTGCCGGTTTCCAGCTC
C-L111Pr	GAGCTGGAAACCGGCAGCGGCAC

C-L116Pf	GTTTCCAGCTCTCCGCTGTTTTATATC
C-L116Pr	GATATAAAACAGCGGAGAGCTGGAAAC
C-L116Rf	GTTTCCAGCTCTCGCCTGTTTTATATC
C-L116Rr	GATATAAAACAGGCGAGAGCTGGAAAC
C-G121Df	GTTTTATATCGACATGGCATTCGCC
C-G121Dr	GGCGAATGCCATGTCGATATAAAAC
C-F130Sf	CTTTGTGGTCTCTCCGCTGGC
C-F130Sr	GCCAGCGGAGAGACCACAAAG
C-P131Lf2	CTTTGTGGTCTTTCTGCTGGCATTTGGC
C-P131Lr2	GCCAAATGCCAGCAGAAAGACCACAAAG
C-A133Vf	CTTTCCGCTGGTGTTTGGCTTC
C-A133Vr	GAAGCCAAACACCAGCGGAAAG
C-L137Hf	CATTTGGCTTCCATGCCAATACC
C-L137Hr	GGTATTGGCATGGAAGCCAAATG
C-A141Ef	CTTGCCAATACCGAGCCGGAAGGG
C-A141Er	CCCTTCCGGCTCGGTATTGGCAAG
C-G144Rf	GCGCCGGAACGCGTGCAGGTATC
C-G144Rr	GATACCTGCACGCGTTCCGGCGC
C-V145Ef	CGGAAGGGGAACAGGTATCCAC
C-V145Er	GTGGATACCTGTTCCCCTTCCG
C-V147Ef	GAAGGGGTGCAGGAATCCACCGAC
C-V147Er	GTCGGTGGATTCCTGCACCCCTTC
C-S148Pf	GTGCAGGTACCGACCGACATC
C-S148Pr	GATGTCGGTCGGTACCTGCAC
C-D150Gf	CAGGTATCCACCGGCATCGCCAGC
C-D150Gr	GCTGGCGATGCCGGTGGATACCTG
C-D150Vf	CAGGTATCCACCGTCATCGCCAGC

C-D150Vr	GCTGGCGATGACGGTGGATACCTG
C-I151Tf	GTATCCACCGACACCGCCAGCTATTTAAG
C-I151Tr	CTTAAATAGCTGGCGGTGTCGGTGGATAC
C-Y154Hf	CATCGCCAGCCATTTAAGCTTC
C-Y154Hr	GAAGCTTAAATGGCTGGCGATG
C-F157Yf	GCTATTTAAGCTACGTTATGGCGCTG
C-F157Yr	CAGCGCCATAACGTAGCTTAAATAGC
C-A160Vf	GCTTCGTTATGGTGCTGTTTATGG
C-A160Vr	CCATAAACAGCACCATAACGAAGC
C-M163Kf	GCGCTGTTTAAGGCGTTTGGTG
C-M163Kr	CACCAAACGCCTTAAACAGCGC
C-S168Pf	CGTTTGGTGTCCCGTTTGAAGTGC
C-S168Pr	GCACTTCAAACGGGACACCAAACG
C-D188Vf	CTCGCCAGAAGTCTTACGCAAAAAACG
C-D188Vr	CGTTTTTTGCGTAAGACTTCTGGCGAG
C-V198Df	GTATGTGCTGGATGGTGCATTCG
C-V198Dr	CGAATGCACCATCCAGCACATAC
C-G204Rf	GCATTCGTTGTCCGCATGTTGCTG
C-G204Rr	CAGCAACATGCGGACAACGAATGC
C-M205Kf	GTTGTCGGGAAGTTGCTGACG
C-M205Kr	CGTCAGCAACTTCCCGACAAC
C-M205Rf	GTTGTCGGGAGGTTGCTGACG
C-M205Rr	CGTCAGCAACCTCCCGACAAC
C-P209Lf	GTTGCTGACGCTGCCGGATGTC
C-P209Lr	GACATCCGGCAGCGTCAGCAAC
C-P210Rf	GCTGACGCCGCGGGATGTCTTC
C-P210Rr	GAAGACATCCCGCGGCGTCAGC

C-S214Pf	GATGTCTTCCCGCAAACGCTG
C-S214Pr	CAGCGTTTGCGGGAAGACATC
C-Q215Rf	GTCTTCTCGCGCACGCTGTTG
C-Q215Rr	CAACAGCGTGCGCGAGAAGAC
C-A219Ef	CAAACGCTGTTGGAGATCCCGATG
C-A219Er	CATCGGGATCTCCAACAGCGTTTG
C-L225Pf	GATGTACTGTCCGTTTGAAATCGG
C-L225Pr	CCGATTTCAAACGGACAGTACATC
C-A47del1	GGATACCAGGTGATAGATGTC
C-A47del2	CCATTGATCAAGCAGTTGCCG
C-G56del1	GCAGTTGCCGCAATCAACGATGATC
C-G56del2	GATCATCGTTGATTGCGGCAACTGC
C-A61del1	GGTTCAACGATGATCACCGACGTGGCC
C-A61del2	GGCCACGTCGGTGATCATCGTTGAACC
C-P71del1	GCCGTTCTTTACGATCAAGCTGACC
C-P71del2	GGTCAGCTTGATCGTAAAGAACGGC
C-Y42del1	GCCAATGACATCCACCTGGTATCC
C-Y42del2	GGATACCAGGTGGATGTCATTGGC
C-A47del3	CACCTGGTATCCCCATTGATCAAG
C-A47del4	CTTGATCAATGGGGATACCAGGTG
C-K51del1	GCGCCATTGATCCAGTTGCCGCAAG
C-K51del2	CTTGCGGCAACTGGATCAATGGCGC
C-P54del1	GATCAAGCAGTTGCAAGGTTCAACG
C-P54del2	CGTTGAACCTTGCAACTGCTTGATC
C-T58del1	CCGCAAGGTTCAATGATCGCCACC
C-T58del2	GGTGGCGATCATTGAACCTTGCGG
C-T62del1	CAACGATGATCGCCGACGTGGCCTC

C-T62del2	GAGGCCACGTCGGCGATCATCGTTG
C-A65del1	GCCACCGACGTGTCGCCGTTCTTTAC
C-A65del2	GTAAAGAACGGCGACACGTCGGTGGC
C-P67del1	GACGTGGCCTCGTTCTTTACGCCG
C-P67del2	CGGCGTAAAGAACGAGGCCACGTC
C-T70del1	CGCCGTTCTTTCCGATCAAGCTG
C-T70del2	CAGCTTGATCGGAAAGAACGGCG
C-K73del1	CTTTACGCCGATCCTGACCTTTATG
C-K73del2	CATAAAGGTCAGGATCGGCGTAAAG
C-Q55G56del1	CAAGCAGTTGCCGTCAACGATGATC
C-Q55G56del2	GATCATCGTTGACGGCAACTGCTTG
C-G56S57del1	CAGTTGCCGCAAACGATGATCGCC
C-G56S57del2	GGCGATCATCGTTTGCGGCAACTG
C-A133del1	GTCTTTCCGCTGTTTGGCTTCCTTG
C-A133del2	CAAGGAAGCCAAACAGCGGAAAGAC
C-N139del1	GGCTTCCTTGCCACCGCGCCGGAAG
C-N139del2	CTTCCGGCGCGGTGGCAAGGAAGCC
C-P142del1	GCCAATACCGCGGAAGGGGTGCAG
C-P142del2	CTGCACCCCTTCCGCGGTATTGGC
C-Q146del1	CCGGAAGGGGTGGTATCCACCGAC
C-Q146del2	GTCGGTGGATACCACCCCTTCCGG
C-A152del1	CCACCGACATCAGCTATTTAAGC
C-A152del2	GCTTAAATAGCTGATGTCGGTGG
C-A160del1	GCTTCGTTATGCTGTTTATGGCG
C-A160del2	CGCCATAAACAGCATAACGAAGC
C-insA42f	CAATGACATCGCATATCACCTGGTATC
C-insA42r	GATACCAGGTGATATGCGATGTCATTG

C-insA47f	CTGGTATCCGCGGCACCATTGATCAAG
C-insA47r	CTTGATCAATGGTGCCGCGGATACCAG
C-insA51f	GCCATTGATCGCCAAGCAGTTGCCGC
C-insA51r	GCGGCAACTGCTTGGCGATCAATGGC
C-insA54f	GATCAAGCAGTTGGCCCCGCAAGGTTC
C-insA54r	GAACCTTGCGGGGCCAACTGCTTGATC
C-insG56f	GTTGCCGCAAGGTGGTTCAACGATGATC
C-insG56r	GATCATCGTTGAACCACCTTGCGGCAAC
C-insGG56f	GTTGCCGCAAGGTGGTGGTTCAACGATG
C-insGG56r	CATCGTTGAACCACCACCTTGCGGCAAC
C-insA58f	CGCAAGGTTCAGCAACGATGATCGCC
C-insA58r	GGCGATCATCGTTGCTGAACCTTGCG
C-insA61f	CAACGATGATCGCAGCCACCGACGTG
C-insA61r	CACGTCGGTGGCTGCGATCATCGTTG
C-insA65f	CCACCGACGTGGCAGCCTCGCCGTTC
C-insA65r	GAACGGCGAGGCTGCCACGTCGGTGG
C-insA67f	GACGTGGCCTCGGCACCGTTCTTTACG
C-insA67r	CGTAAAGAACGGTGCCGAGGCCACGTC
C-insA70f	CCGTTCTTTGCAACGCCGATCAAGC
C-insA70r	GCTTGATCGGCGTTGCAAAGAACGG
C-insA133f	GTCTTTCCGCTGGCAGCATTTGGCTTC
C-insA133r	GAAGCCAAATGCTGCCAGCGGAAAGAC
C-insA139f	GCTTCCTTGCCGCAAATACCGCGCC
C-insA139r	GGCGCGGTATTTGCGGCAAGGAAGC
C-insA142f	CCAATACCGCGGCACCGGAAGGGGTG
C-insA142r	CACCCCTTCCGGTGCCGCGGTATTGG
C-insA146f	CGGAAGGGGTGGCACAGGTATCCAC

C-insA146r	GTGGATACCTGTGCCACCCCTTCCG
C-insA152f	CACCGACATCGCAGCCAGCTATTTAAG
C-insA152r	CTTAAATAGCTGGCTGCGATGTCGGTG
C-insA160f	GCTTCGTTATGGCAGCGCTGTTTATG
C-insA160r	CATAAACAGCGCTGCCATAACGAAGC

Table S3. Oligonucleotides used in this study.

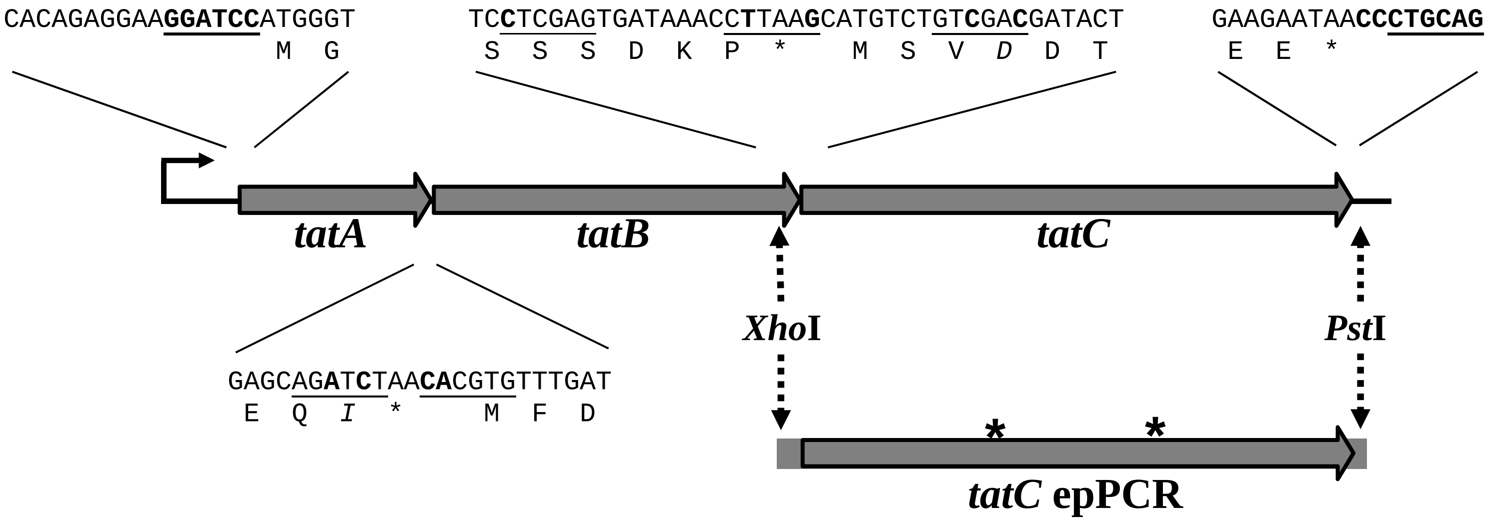

Kneuper *et al*. Fig S1

A

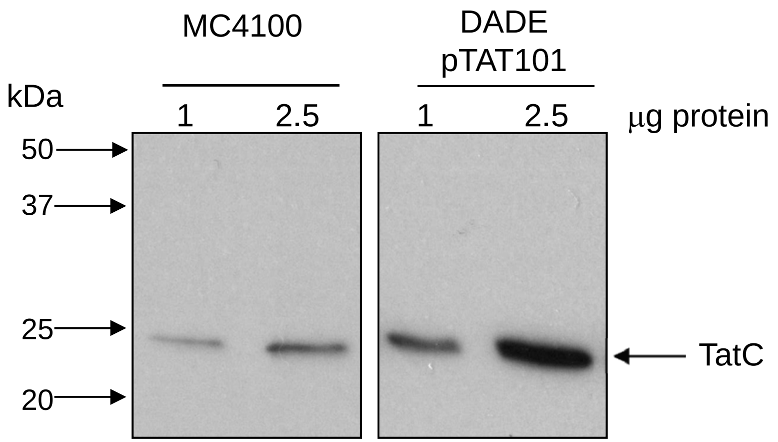

B

	MC4100		DADE pTAT101	
μg total protein	1	2.5	1	2.5
Image J area value	2605	8658	10502	30867

Kneuper *et al*. Fig S2

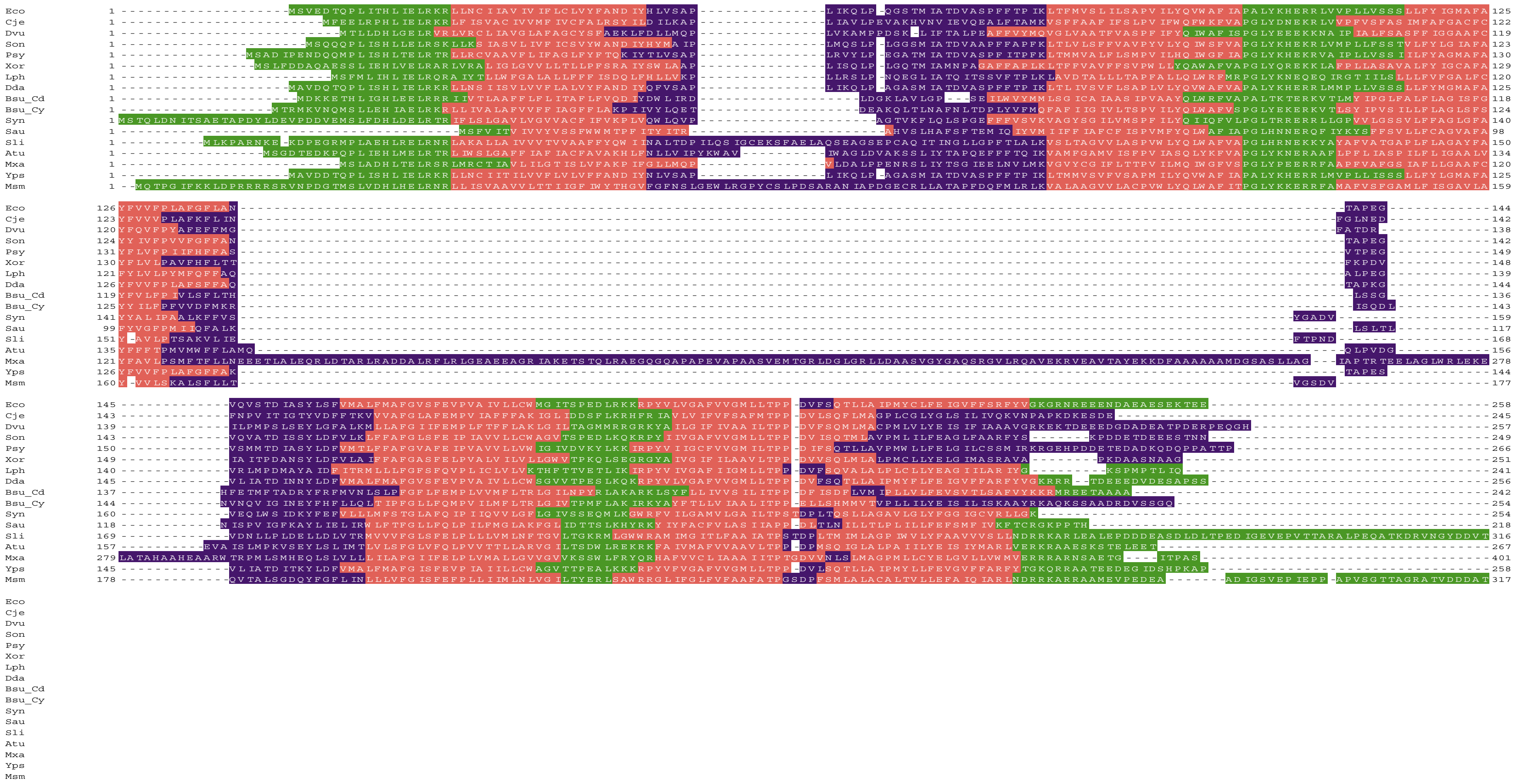

Kneuper *et al*. Fig S3
